# Integrated Microbiome and Metabolomics Insights into Meat Quality Changes in Rice-Field Eel Slices During Refrigeration Storage: Effects of ε-Polylysine, Vitamin C, Epigallocatechin Gallate, and Phloretin

**DOI:** 10.3390/foods14132236

**Published:** 2025-06-25

**Authors:** Liu Shi, Lifeng Yang, Juan You, Wenjin Wu, Guangquan Xiong, Lan Wang, Tao Yin

**Affiliations:** 1 Key Laboratory of Cold Chain Logistics Technology for Agro-Product, Ministry of Agriculture and Rural Affairs, Institute of Agro-Product Processing and Nuclear Agricultural Technology, Hubei Academy of Agricultural Sciences, Wuhan 430064, China; shiliu@hbaas.com (L.S.); ylifeng2022@163.com (L.Y.); wuwenjin@hbaas.com (W.W.); xiongguangquan@163.com (G.X.); 2 College of Food Science and Technology, Huazhong Agricultural University, Wuhan 430070, China; juanyou@mail.hzau.edu.cn; 3 National R&D Branch Center for Conventional Freshwater Fish Processing (Wuhan), Wuhan 430070, China; 4 College of Food and Quality Engineering, NanNing University, Nanning 530200, China

**Keywords:** muscle quality, biomarker, 16S rRNA sequencing, metabolic pathway, myoglobin

## Abstract

Rice-field eel (*Monopterus albus*) slices, an important aquatic product in Southeast Asia, are prone to spoilage and deterioration during cold chain storage. In this study, the effects of a composite preservative (ε-polylysine, Vitamin C (Vc), epigallocatechin gallate (EGCG), and phloretin) on the muscle quality (color, texture, water holding capacity (WHC)) of rice-field eel slices during refrigeration storage at 4 °C for up to 7 days was investigated, and the underlying mechanism was elucidated by the integrated microbiome and metabolomics, in addition to Elisa and Low-Field Nuclear Magnetic Resonance (LF-NMR). After 7 days of storage, the WHC, shear force, and a* decreased by 11.39%, 34.37%, and 49.20% in treated samples, and by 19.18%, 38.38%, and 54.87% in control samples, respectively. The addition of the composite preservative significantly increased Hexokinase, Pyruvate kinase, and Creatine kinase, while it decreased the total viable count (TVC), total volatile basic nitrogen (TVB-N), thiobarbituric acid reactive substance (TBARS), and Lactic acid. Preservative treatment maintained the moisture content of the eel slices during storage and prevented bright red oxymyoglobin from transforming into brown metmyoglobin. Microbiota composition (especially *Pseudomonas*) and metabolic pathways (including amino acid and its metabolites, nucleotide and its metabolite, and organic acid and its derivatives, etc.) were obviously altered by the preservative treatment. *Pseudomonas*, tryptophan-aspartic acid (Trp-Asp), D-Glucose 6-phosphate, Succinic Acid, Biliverdin 1, 5-Diaminopentane, and Tyramine, etc., are potential biomarkers for the quality changes of eel slices during refrigeration. These findings provide an in-depth understanding of the improvement of the eel slice quality during refrigeration storage by the composite preservative.

## 1. Introduction

Rice-field eel (*Monopterus albus*) is a tropical freshwater fish (Figure S1A) belonging to the *Hemibranchia* Order, *Hemibranchidae* Family, and *Monopterus* Genus [1]. Rice-field eel is mainly distributed in Southeast Asian countries, such as China, Japan, North Korea, and Thailand. The total production of rice-field eel raised in China in 2023 was 355, 203 tons [2]. At present, the eel was mainly processed into a fresh sliced product. This product is prone to spoilage and deterioration during refrigeration storage within 3 days (Figure S1B,C), manifested as color browning, odor generation, meat dispersion, and decreased water holding capacity (WHC) [3], which reduces the edible quality, safety, and commercial value of the eels.

Adding preservatives is an effective method for preserving fish products. Antibacterial agents are the most common components in preservatives, which extend the shelf life of fish products by inhibiting the growth of microorganisms. ε-polylysine (ε-PL), a monomeric polymer containing 25–30 lysine residues, is a natural antibacterial agent. Due to its wide antibacterial spectrum and good stability compared to other antibacterial agents, ε-polylysine has entered the commercial market of more and more countries in recent years. According to the research results of Li et al. [4] and Qian et al. [5], ε-polylysine has great potential for application in the preservation of fish products.

During the refrigeration process, physiological programs such as cell apoptosis are initiated due to oxidative stress and other factors, which affect the texture, WHC, and other edible qualities [6,7]. Meanwhile, biomolecules such as lipids can produce unpleasant flavors after oxidation [8]. While antimicrobial agents such as ε-polylysine effectively suppress microbial proliferation, their capacity to mitigate oxidative deterioration—a major spoilage pathway—remains constrained. Therefore, adding an antioxidant is also an important measure to prevent the quality deterioration of fish products in refrigeration storage. Common antioxidants used in the preservation of fish products include Vc, epigallocatechin gallate (EGCG), phloretin (Pht), and others [9]. Due to the varied chemical structures and antioxidant mechanisms, different antioxidants have a synergistic effect, thus maintaining the quality of fish meat during storage [10,11].

Lambrianidi et al. [3] studied the effects of chitosan and oregano oil on the quality of eel slices during 4 °C refrigeration and found that adding chitosan and oregano oil significantly extended the shelf life compared to adding chitosan alone. In recent years, scholars have also confirmed in their research on the storage of sea bass and shrimp that ε-polylysine and antioxidants (essential oil, gallic acid, etc.) have a synergistic effect on the preservation of aquatic products [4,5]. At present, there is no report on the effect of combining ε-polylysine with different antioxidants (Pht, Vc, and EGCG) on the quality changes of eels during refrigeration.

Omics analysis can significantly enhance our comprehension of the intricate mechanisms governing muscle quality deterioration during refrigeration storage. Microbial and metabolite changes are important factors affecting muscle quality [12,13,14]. Previously, a combined approach of microbiome and metabolomics has been used by Guo et al. [15] for investigating the quality changes in mutton during frozen storage at −20 °C. They reported that the color of mutton muscle darkened, and the content of 1, 2-Dioleoyl-sn-glycero-3-phosphoethanolamine, C20:3n-6, C23:0, Indole-3-acrylic acid, and the other metabolites was significantly altered during frozen storage. The metabolic pathways were mainly enriched in nucleotide metabolism, purine metabolism, and an amino acid metabolic pathway. Additionally, the abundance of bacteria, including *Aeromonas*, *Brachymonas*, *Corynebacterium*, and *Steroidobacter*, increased significantly. A significant correlation was found between these metabolites and microorganisms in mutton. The spoilage dynamics vary considerably across raw food materials, where microbial community succession and the accumulation of critical metabolites are highly specific to the material type. However, the changes in microbial and metabolite composition during the refrigeration storage of eel slices, as well as how they affect muscle quality, are still unclear.

Therefore, in this study, rice-field eel slices were refrigerated at 4 °C for different periods of time to study changes in muscle quality, biochemical indicators, microorganisms, and metabolites. Simultaneously, the effects of combining ε-polylysine with three antioxidants (Vc, EGCG, and Pht) on improving the quality of the eel slices during storage were investigated in order to provide a solution for extending their shelf life.

## 2. Material and Methods

### 2.1. Material

Rice-field eel (*Monopterus albus*) (200 ± 50 g/tail) was purchased from the Baishazhou wholesale market of aquatic products in Wuhan (China). ε-Polylysine was purchased from Yuanye Biotechnology Co., Ltd. (Shanghai, China). Antioxidants, including Pht, VC, and EGCG, were purchased from Zesheng Technology Co., Ltd. (Hefei, China), China National Pharmaceutical Group Chemical Reagent Co., Ltd. (Wuhan, China), and Bomei Biotechnology Co., Ltd. (Hefei, China), respectively. The kits of glycogen (GLY), lactate (LA), creatine kinase (CK), pyruvate kinase (PK), lactate dehydrogenase (LDH), and hexokinase (HK) were purchased from Jiancheng Bioengineering Institute (Nanjing, China). Other chemical reagents were purchased from China National Pharmaceutical Group Chemical Reagent Co., Ltd. (Wuhan, China).

### 2.2. Preparation and Treatment of Eel Slice

A total of 50 kg of fresh live eels were purchased from a local market (Wuhan, China) and transported alive to the laboratory within 30 min. The eels were placed in plastic bags containing a small amount of water to maintain surface moisture, and were kept at room temperature during transport. Skilled operators then processed the eels using a slaughtering machine (Model A01S02, Jingzhou Jichuang Electromechanical Technology Co., Ltd., Jingzhou, China). The procedure involved insertion through the machine’s inlet, followed by abdominal dissection, removal of bones, and severing of the nervous system. The processed eels were then discharged through the outlet. The average slaughtering speed was approximately 2 s per eel. All experimental procedures were conducted in accordance with the research protocol approved by the Institutional Animal Care and Use Committee of Huazhong Agricultural University (Wuhan, Hubei), with the ethical code of HZAUFI-2025-0001. The carcass was cut into slices about 4 cm in length and 3 cm in width (Figure S1B), among which samples with a uniform size were selected for storage tests. The slices were soaked in a composite preservative containing 0.01% ε-polylysine, 0.4% V_C_, 0.3% EGCG, and 0.6% Pht for 10 min. The formula of this composite preservative was optimized through orthogonal experiments based on our preliminary research (data not published). The orthogonal experiment (L9(3^3^)) tested ε-polylysine concentrations at 0.01%, 0.015%, and 0.02%; Vc, EGCG, and phloretin concentrations at 0.1%, 0.3%, and 0.5%; there was a fixed immersion time of 10 min. The optimization criteria were the comprehensive preservation quality on day 7, as follows: total bacterial count, meat color stability (R630/580), and sensory score. After vacuum packaging the slices in a polyethylene bag, they were stored at 4 °C for up to 7 days. The slices of the control group (CK) were immersed in deionized water for 10 min to ensure they had the same hydration conditions. The treatment group and control group are labeled with T and CK, respectively, and the number combined with T or CK represents the storage days. All of the sampling procedures were approved by the Animal Care and Use Committee of Hubei Academy of Agricultural Sciences.

### 2.3. Determination of WHC

The determination of WHC was based on Zhu et al.’s [16] method, with slight modifications. Two grams of eel muscle were wrapped with four layers of filter paper, and then further wrapped with gauze. The sample was centrifuged at 4 °C and 4000 r/min for 10 min. The WHC is the ratio of the moisture content of the sample after centrifugation to that before centrifugation.

### 2.4. Determination of Shear Force

Determining the shear force was carried out using Lin et al.’s [17] methods. The eel muscle was cut into 3 cm × 1 cm pieces, and then the XT 2i/50 texture analyzer (Minolta (China) Investment Co., Ltd., Shanghai, China) was used to determine shear force. The testing conditions were an HDP/BS probe, a force arm of 25 kg, a pre-test rate of 5.0 mm/s, a mid-test rate of 1.0 mm/s, a post-test rate of 5.0 mm/s, and a compression deformation of 50%.

### 2.5. Determination of Color

The CM-2500c spectrophotometer (Minolta (China) Investment Co., Ltd., Shanghai, China) was used to measure the brightness (L*), red/green degree (a*), yellow/blue degree (b*), chromaticity value (C*), chromaticity angle (H*), and R630/580 value of the eel muscle. The C* value represents the red intensity of the meat sample, and the H* value represents the chromaticity angle [18]. R630/580 represents the stability of color, with a higher value indicating a more stable color [19].

### 2.6. Determination of pH, TVB-N, TBARS, and TVC

Two grams of ground eel muscle were added to 18 mL of deionized water, centrifuged at 4000 r/min for 10 min. The pH of the supernatant was measured using the FG2-B portable pH meter (Mettler Toledo Instruments Co., Ltd., Shanghai, China). For TVB-N analysis, 2 g of eel muscle was homogenized with 20 mL distilled water and standing for 30 min at 4 °C. The solution was filtered, and the filtrate was collected. The semi-micro Kjeldahl nitrogen method was applied to determine the TVB-N of the eel muscle. TBARS content was measured using a commercial reagent kit (A003-1, Jiancheng Bioengineering, China) following the manufacturer’s protocol. Briefly, samples were homogenized in 7.5% (*w*/*v*) trichloroacetic acid containing 0.1% EDTA, reacted with thiobarbituric acid at 95 °C for 40 min, and the absorbance was read at 532 nm after centrifugation. Furthermore, for the total viable colonies (TVC) detection, 3 g samples were aseptically minced, homogenized in 27 mL sterile 0.85% saline, and vortex-mixed. After decimal dilution, 1 mL aliquots were plated in triplicate on a Plate Count Agar (PCA) and incubated at 30 °C for 48 h. Colonies were counted and expressed as log CFU/g.

### 2.7. Determination of Glycogen and Lactate Contents, and Hexokinase, Pyruvate Kinase, Lactate Dehydrogenase, and Creatine Kinase Activities

Glycogen content (GLY) and the lactate concentration (LA) were quantified using enzymatic colorimetric kits, while the enzyme activities of hexokinase (HK), pyruvate kinase (PK), lactate dehydrogenase (LDH), and creatine kinase (CK) were determined via corresponding reagent kits. All measurements used reagent kits (Nanjing Jiancheng Bioengineering Institute, China) and a Spark microplate reader (Tecan, Grödig, Austria). The wavelengthes for the GLY, LA, HK, PK, LDH and CK determination were 620 nm, 530 nm, 340 nm, 340 nm, 440 nm and 660 nm, respectively. HK activity is expressed in units of nmol/min/mg (tissue), where one unit (U) is defined as the amount of enzyme that produces 1 nmol of NADPH per minute under conditions of 37 °C for 20 min. The activity of PK is expressed in the unit ‘U/gprot’ (tissue). Here, one unit (U) is defined as follows: under conditions of 37 °C and pH 7.6, for every gram of tissue protein, 1 µmol of PEP is converted into pyruvate per minute, which represents one enzymatic activity unit. The activity of LDH is expressed in the unit of ‘U/gprot’ (tissue). Here, one unit (U) is defined as follows: 37 °C, 15 min of reaction with the matrix in 1 g of tissue protein, and 1 µmol of pyruvic acid produced is regarded as 1 unit of activity. The activity of CK is expressed in the form of ‘U/mgprot’ (tissue). Here, one unit (U) is defined as follows: 45 °C, 15 min of reaction with the matrix on 1 g of tissue protein, and 1 µmol of creatine kinase produced in the reaction system represents 1 unit of activity. The specific operation and result calculation of these attributes were carried out according to the instructions of each reagent kit.

### 2.8. Determination of Myoglobin Content

The determination of myoglobin content was carried out according to the method of Krzywlckt, K. [20], with slight modifications. Five grams of the sample were added with 25 mL of phosphate buffer solution (0.04 mol/L, pH 6.8), homogenized with ice incubation at 10,000 r/min for 2 min, and standing at 4 °C for 1 h. Then it was centrifuged at 4500 g for 20 min at 4 °C. The supernatant was filtered and diluted to 25 mL with the same phosphate buffer solution. The absorbance values at 525, 545, 565, and 572 nm were measured using a UH5300 UV-visible spectrophotometer (Hitachi, Ltd., Tokyo, Japan). The relative contents of different forms of the myoglobin, including deoxymyoglobin, oxymyoglobin, and metmyoglobin, were calculated separately.

### 2.9. Analysis of Water Status and Distribution

Analysis of the water status and distribution was followed by the method of Zhao et al. [21]. The eel sample was cut into 1 cm × 1 cm × 1 cm cubes and placed in a nuclear magnetic tube. After the sample temperature reached equilibrium with the ambient temperature, the MI20-025V-I nuclear magnetic resonance analyzer (Newman Analytical Instruments Co., Ltd., Suzhou, China) was used to collect T_2_ signals (spin–spin relaxation time) and conduct magnetic resonance imaging (MRI) tests. In the MRI image, red color corresponds to high hydrogen proton density, and blue color corresponds to low hydrogen proton density.

### 2.10. Microbiota Analysis

Microbial diversity analysis referred to the method by Guo et al. [15]. After extracting DNA from eel muscle, a PCR amplification of 16S rRNA gene was performed (ABI GeneAmp^®^ 9700 model, Thermo Fisher Scientific (China) Co., Ltd., Shanghai, China). The PCR experiment employed a reaction system of TransGen AP221-02 and TransStart Fastpfu DNA Polymerase (20 μL), as follows: 5 times of FastPfu Buffer, 2.5 mM dNTPs, Forward Primer (5 μM), Reverse Primer (5 μM), FastPfu Polymerase, BSA, Template DNA. PCR reaction parameters were 95 °C for 3 min, cycle number 32 (95 °C and 30 s, 60 °C and 30 s, 72 °C and 45 s), 72 °C for 10 min. After adding the standard enrichment of PCR products, 10,000 sequences per sample were determined. The sample was denatured with 0.1 mol/L NaOH solution to obtain single-stranded DNA fragments. A sequencing library was constructed, and high-throughput sequencing was performed on the 16s-338F-806R Miseq platform of Meiji Biomedical Technology Co., Ltd. (Shanghai, China). Statistical analysis on the obtained sequencing data was performed using MOTHUR, EXCEL, and SPSS software.

### 2.11. Untargeted Metabolomics Profiling

Using Peng et al.’s methods [22], liquid chromatography–tandem mass spectrometry (UPLC-MS/MS) was employed to study the metabolomics profiling of eel muscle. The sample was taken out from the −80 °C freezer and thawed on ice until it could be cut easily (all subsequent operations were required to be performed on ice). The sample was chopped and mixed prior to the homogenization with a ball mill (30 Hz) for 20 s. The homogenized sample was centrifuged at 4 °C. A 70% methanol–water extraction solution containing an internal standard was added to the supernatant. After oscillation, it was centrifuged at 4 °C. Three hundred μL of the supernatant was transferred and stood at −20 °C for 30 min, and then it was centrifuged again. Three hundred μL of the supernatant was transferred into the inner tube of the injection bottle for machine analysis. The data acquisition instrument system mainly included ultra-performance liquid chromatography (UPLC) (ExionLC AD, Shanghai, China, https://sciex.com.cn/) and tandem mass spectrometry ((MS/MS) (QTRAP)^®^, https://sciex.com.cn/). Unsupervised principal component analysis (PCA) was used to analyze the overall metabolite differences between samples in each group and the variability between samples within the group. Variables with a variable important in projection (VIP) value greater than 1 were considered differential variables. A screening and identification differential was performed along with a *t*-test (*p* < 0.05). The screened differential expression metabolites (DEM) were mapped to the KEGG network database (https://www.genome.jp/kegg/pathway.html, accessed on 15 May 2024), and the metabolites with the highest matching degree were selected and confirmed.

### 2.12. Statistical Analysis

The preparation of the samples was repeated twice. Three biological samples were used for microbial diversity analysis, and six biological samples were used for metabolomics analysis. Muscle quality and biochemical indicators were measured in parallel at least twice. Data from muscle quality and biochemical indicators were analyzed using one-way analysis of variance (ANOVA) followed by Tukey’s post hoc test for group comparisons with SPSS 26.0 (IBM, Armonk, NY, USA), with significance defined at *p* < 0.05. GraphPad Prism 5.0 software was used for drawing the figures. The correlation between muscle quality and biochemical indicators, and microorganisms and metabolites, is presented using an integrated heat-map and network diagram, which was drawn by ggcor 0.98.1 R (4.3.2).

## 3. Results

### 3.1. Changes in Meat Quality Under Refrigeration Storage

#### 3.1.1. WHC, Shear Force, and Color

**Figure 1:**
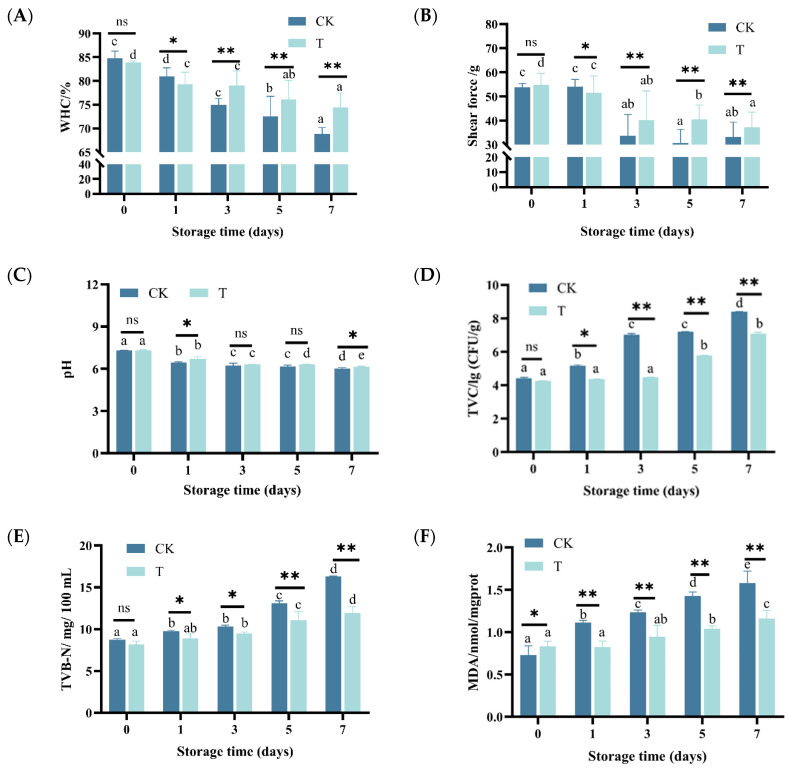
Changes in WHC, shear force, pH, TVC, TVB-N, and TBARS of rice-field eel slices during refrigeration storage, as affected by the addition of ε-polylysine and antioxidants. (**A**) WHC—water holding capacity, (**B**) shear force, (**C**) pH, (**D**) TVC, (**E**) TVB-N, (**F**) TBARS. CK—control group, T—treatment group with the addition of ε-polylysine and antioxidants. The numbers indicate storage day. Different lowercase letters indicate significant differences (*p* < 0.05) in the samples under different storage times. ns indicates no significance, * indicates *p* < 0.05, ** indicates *p* < 0.01.

As shown in Figure 1A,B, the WHC and shear force of eel slices showed a decreasing trend with prolonged storage time (*p* < 0.05). After storage for more than 3 days, the WHC and shear force of the T group were significantly higher than those of the CK group (*p* < 0.01). On the 7th day of storage, the WHC of the CK and T group decreased by about 19.18% and 11.39%, respectively, and the shear force decreased by 38.38% and 34.37%, respectively. The color parameters of stored eel slices are listed in Table 1. As time went on, a* and R630/580 gradually decreased (*p* < 0.05), while L*, b*, C*, and H* showed a trend of first increasing and then decreasing (*p* < 0.05). During storage, the L*, a*, C*, H*, and R630/580 of the T group of eel slices were higher than those of the CK, indicating that the preservative treatment can effectively maintain the bright and red color of eel slices.

#### 3.1.2. pH, TVC, TVB-N, MDA, and TBARS

After one day of storage, the pH of eel slices significantly decreased, with the pH of the CK and T groups decreasing from 7.31 and 7.29 to 6.71 and 6.45, respectively (Figure 1C). With the extension of the storage time, the pH of the CK and T groups decreased slowly (*p* < 0.05), reaching 6.15 and 6.02 at day 7, respectively. While the pH decrease was largely similar between the CK and T groups (*p* < 0.05 over time), the T group consistently exhibited slightly lower pH values throughout storage. It is worth noting that in the early stage of pH decline, it is mainly driven by the post-mortem glycolysis of the muscle tissue and the endogenous enzyme activity (ATP degradation, lactate production), while in the later stage, it is mainly caused by acid production by microorganisms [23]. As shown in Figure 1D–F, TVC, TVB-N, and TBARS gradually increased with prolonged storage time (*p* < 0.05). During storage, the TVC, TVB-N, and TBARS of the T group were significantly lower than those of the CK group (*p* < 0.05), indicating that preservative treatment can effectively inhibit microbial decay and oxidation. This also explains why, in the later stage of storage (7 d), the pH value of group T was slightly higher than that of the control group.

### 3.2. Differences in Water Status and Distribution Under Refrigeration Storage

**Figure 2:**
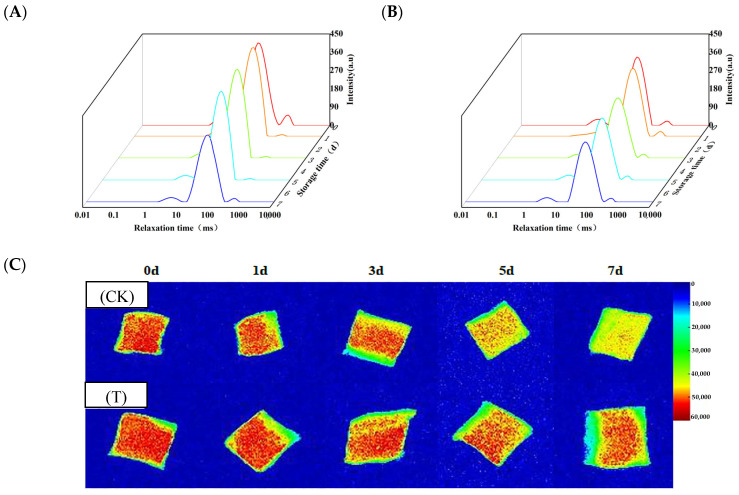
Changes in water status and distribution of rice-field eel slices during refrigeration storage as affected by the addition of ε-polylysine and antioxidants. (**A**,**B**) Water status of the CK and T groups, respectively, (**C**) water distribution. CK—control group, T—treatment group with the addition of ε-polylysine and antioxidants; the numbers indicate storage day.

Figure 2A,B show the typical water status of aquatic products. The T_2_ relaxation time distribution of eel slices peaked at 0–10 ms, 10–100 ms, and 100–1000 ms, corresponding to water tightly bound to macromolecules (P_21_), immobilized water inside myofibrils (P_22_), and free water distributed outside myofibrils (P_23_), respectively. With the extension of storage time, the P_21_, P_22_, and P_23_ of the two groups of slices showed a fluctuating trend. The fluctuating trends could be attributed to the combined effects of microbial activity, enzymatic changes, and water migration during storage. Except for the 5th day, the P_23_ of the T group was lower than that of the CK group, suggesting that preservative treatment can reduce water leakage from the muscles.

As shown in Figure 2C, during the early stage of storage (3 days), most areas of the images of the two groups were red in color, with only the edge areas being yellow. As the storage time prolonged, the area of the red region gradually decreased, while the area of the yellow and green regions gradually increased. Compared to the CK group, the red area of the T group was apparently larger during storage. Especially on the 7th day, more than half of the area in the T group samples was displayed in red, while only sporadic red dots were visible in the CK group. Therefore, the preservative treatment can maintain the moisture content of eel slices during storage.

### 3.3. Changes in Biochemical Indicators Under Refrigeration Storage

#### 3.3.1. GLY, LA, HK, PK, LDH, and CK

**Figure 3:**
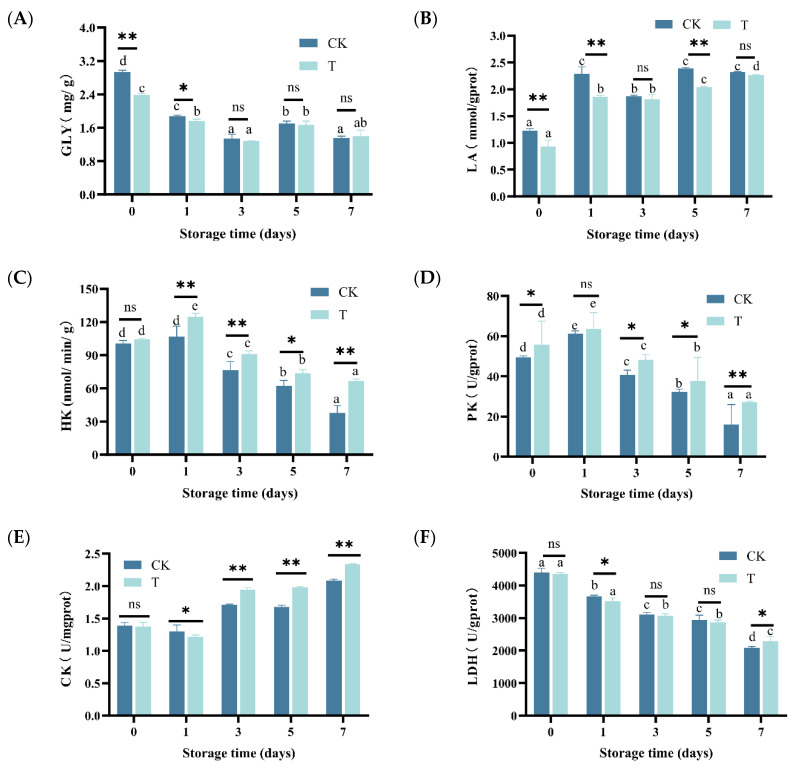
Changes in GLY, LA, HK, PK, CK, and LDH of rice-field eel slices during refrigeration storage, as affected by the addition of ε-polylysine and antioxidants. (**A**) GLY—glycogen, (**B**) LA—lactic acid, (**C**) HK—hexokinase, (**D**) PK—Pyruvate kinase, (**E**) CK—creatine kinase, (**F**) LDH—lactic dehydrogenase. CK—control group, T—treatment group with the addition of ε-polylysine and antioxidant; the numbers indicate storage day. Different lowercase letters indicate significant differences (*p* < 0.05) among samples with different storage times. ns indicates no significance, * indicates *p* < 0.05, ** indicates *p* < 0.01.

During storage, the GLY content of eel slices showed an overall decreasing trend, and it rapidly decreased in the first 3 days (*p* < 0.05) (Figure 3A). On the contrary, the LA content continued to increase (Figure 3B).

The activity of HK showed a trend of first increasing and then decreasing (Figure 3C), reaching its maximum value on day 1 (106.87 nmol/min/g) and decreasing to its minimum value on day 7 (37.91 mmol/min/g). The activity of PK showed a similar trend to that of HK, with a maximum value of 61.27 U/g prot and a minimum value of 16.16 U/g prot (Figure 3D). As shown in Figure 3E,F, with prolonged storage time, CK activity showed a trend of first decreasing and then increasing (*p* < 0.05), while LDH activity gradually decreased (*p* < 0.05).

As depicted in Figure 3A–E, the GLY content in the T group was higher than that in the CK group during the early storage period (*p* < 0.05), but there was no significant difference between the two after prolonged storage (*p* > 0.05). Except for days 3 and 7, the LA content in group T was significantly lower than that in group CK. Except for the first day, the activity of HK, PK, and CK in the T group was significantly higher than that in the CK group (*p* < 0.05). Compared to GLY, LA, HK, PK, and CK, the difference in LDA between the T group and CK group is relatively small.

#### 3.3.2. Myoglobin

As shown in Figure S2, during the refrigeration storage of the eel slices, the relative content of MetMb in the muscle gradually increased, the relative content of OxyMb gradually decreased, and DeoMb showed fluctuating changes. The trend of changes in different forms of myoglobin in the T group is basically consistent with that in the CK group. Compared to CK, the relative content of OxyMb in group T is relatively higher, while the content of DeoMb and MetMb is relatively lower. Preservative treatment can prevent bright red OxyMb from transforming into brown MetMb, which is consistent with the results of color measurement (Table 1).

### 3.4. Changes in Microbiota Composition Under Refrigeration Storage

**Figure 4:**
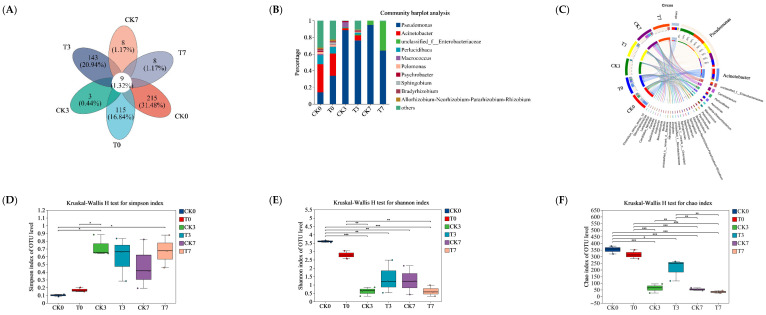

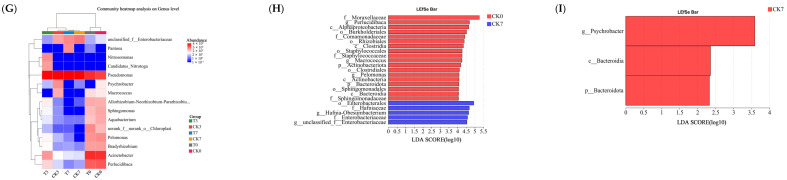
Analysis of microbial diversity of rice-field eel slices during refrigeration storage as affected by the addition of ε-polylysine and antioxidants. (**A**) Venn diagram shows number of unique and shared species in different groups. (**B**) Bar chart of microbial community distribution at the genus level. (**C**) Circos sample and species relationship diagram at genus level. (**D**–**F**) Indicate Shannon, Simpson, and Chao bacterial diversity indexes, respectively. (**G**) Community heat-map analysis on Genus level. (**H**,**I**) The LEfSe analysis between CK0 and CK7 (LDA > 4) from Phyium to genus level, between CK7 and T7 (LDA > 2), respectively. CK—control group, T—treatment group with the addition of ε-polylysine and antioxidants; the numbers indicate storage day. * indicates *p* < 0.05, ** indicates *p* < 0.01, *** indicates *p* < 0.001.

In this study, 16S rRNA sequence analysis was used to investigate the changes in microbiota composition of the eel slices during the refrigeration storage. After sequence alignment using a 97% sequence similarity criterion, a total of 1281 OTUs were identified, including 9 OTUs shared for the six groups. Among them, 251, 3, and 8 OTUs were only identified in the CK group, stored for 0, 3, and 7 days, respectively. Additionally, 115, 148, and 8 OTUs were only found in the CK group, stored for 0, 3, and 7 days, respectively (Figure 4A). According to the overall bacterial community composition (Figure 4B), we found that the primary species of bacteria at the genus level were *Acinetobacter* (33.6%), *Pseudomonas* (14.0%), and *Perlucidibaca* (10.9%) in the CK group before storage. The *Pseudomonas* became dominate at days 3 and 7, with the percentage of community abundance at 88.6% and 94.6%, respectively. Correspondingly, *Acinetobacter* decreased to 2.1% and <1% at days 3 and 7, respectively. Unclassfied_f_*Enterobacteriaceae* with a percentage of 5.1% appeared at day 7. The T group showed a similar percentage of community abundance to the CK group; however, the percentage of *Pseudomonas* was obviously lower during storage. The Circos diagram depicts the relationship between samples and species (Figure 4C). *Proteobacteria* was dominate at the genus level, of which the samples of CK0, CK3, CK7, T0, T3, and T7 contributed 4%, 10%, 27%, 22%, 13%, and 25%, respectively. *Acinetobacter* was the second largest at the genus level, with the percentages of CK0, CK3, CK7, T0, T3, and T7 contributing 46%, 28%, 3%, 8%, 15%, and 0%, respectively.

As shown in Figure 4D–F, the Simpson index of the OUT level increased after storage, while the Shannon and Chao indices decreased, suggesting decreased microbial richness and diversity of microbiota in the eel slices. The alpha diversity (Shannon, Simpson, and Chao1) of the slice microbiota during storage indicated similar bacterial diversity indices between the samples with and without preservation treatment. The community patterns of the six samples were compared using microbial community heat-map analysis (Figure 4G). They can be clustered into two primary groups, namely the group without storage and the group with storage. T7 sample can be clustered with a CK7 sample, CK3 sample, or T3 sample at the sub-group level.

LEfSe analysis was carried out to screen the differential biomarkers between the CK0 and CK7 groups (Figure 4H), and between the CK7 and T7 groups (Figure 4I), from phylum to genus levels. The screening result showed that two Phyla, four Classes, six Orders, six Families, and four Genera could be treated as candidate biomarkers between the CK0 and CK7 groups. Furtheremore, there was 1 Phyla, 1 Class, and 1 Genus between the CK7 and T7 groups. Compared to the CK0 group, the CK7 group showed an obvious predominance of *Enterobacterales*, *Hafniaceae*, *hafnia-Obesumbacterium*, and *Enterobacteriaceae*. According to the LEfSe analysis between the CK7 and T7 groups, *Psychrobacter*, *Bacteroidia*, and *Bacteroidota* were key differential microorganisms affected by the preservative treatment. However, it should be noted that the direct causal relationships between these differential microbiota and meat quality parameters need to be ascertained through future research.

### 3.5. Metabolic Changes Under Refrigeration Storage

#### 3.5.1. Metabolomics Profiling

**Figure 5:**
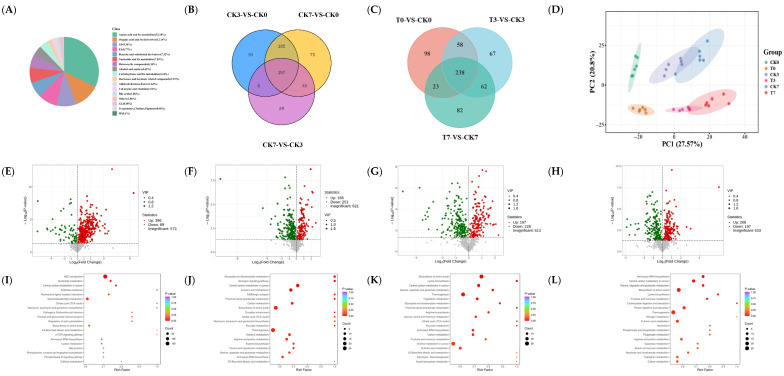
Analysis of metabolomic data in rice-field eel slices during refrigeration storage, as affected by the addition of ε-polylysine and antioxidants. (**A**) Class count of metabolites in all groups. (**B**,**C**) Venn diagram. (**D**) PCA analysis. (**E**–**H**) Volcano plot. (**I**–**L**) Bubble plot for differential metabolites of CK7 vs. CK0, T0 vs. CK0, T3 vs. CK3, and T7 vs. CK7. KEGG pathway analyses of CK7 vs. CK0, T0 vs. CK0, T3 vs. CK3, and T7 vs. CK7. CK—control group, T—treatment group with the addition of ε-polylysine and antioxidants; the numbers indicate storage day.

In this experiment, a total of 1109 metabolites were identified in the eel muscle, including amino acids and their metabolites (32.18%), organic acids and their derivatives (12.14%), glycerophospholipids (9.34%), fatty acyls (8.77%), benzene and its substituted derivatives (7.32%), nucleotides and their metabolites (7.03%), heterocyclic compounds (6.36%), alcohols and amines (4.62%), carbohydrates and their metabolites (4.24%), hormones and hormone-related compounds (1.93%), aldehydes, ketones, esters (1.64%), coenzymes and vitamins (1.16%), bile acids (1.06%), others (1.06%), glycerolipids (0.58%), serotonin, choline, pigments (0.48%), and sphingolipids (0.1%) (Figure 5A).

The Venn diagram shows 357 shared metabolites at different storage times. Different expressed metabolites (DEM) between CK3 and CK0, and between CK7 and CK0, were 382 and 465, respectively. Among them, 342 were the different shared metabolites (Figure 5B). The number of different metabolites in the treatment group and the control group on days 0, 3, and 7 were 417, 425, and 405, respectively, with 238 being shared metabolites (Figure 5C). According to the PCA analysis results, there was no overlap between the CK groups and the T groups (Figure 5D). At the same time, the CK groups were located right above the T groups, indicating that the PC2 axis can explain the impact of preservative treatment. With the extension of storage time, both groups of samples gradually moved to the right, indicating that the PC1 axis can explain the effect of storage time. The samples stored for 3 and 7 days partially overlapped, but both were separated from the samples stored for 0 days.

The DEM with a VIP > 1 and *p*-value < 0.05 are visually displayed in the form of a volcanic plot (Figure 5E–H). As compared to CK0, 396 and 69 metabolites were upregulated and downregulated in the CK7 sample, respectively (Figure 5E). With respect to CK0, CK3, and CK7, 166, 197, and 208 metabolites were upregulated in the samples of T0, T3, and T7, respectively. Additionally, 251, 228, and 197 metabolites were downregulated (Figure 5F–H). DEMs with an absolute value of Log2FC > 1 were further screened out and showed in Tables S2–S5. Compared to CK0, 140 amino acids and their metabolites, 17 nucleotides and their metabolites, 14 organic acids and their derivatives, 14 GPs, 8 FAs, 3 carbohydrates and their metabolites, 1 benzene and substituted derivatives, 3 alcohol and amines, and 3 bile acids were upregulated. Furthermore, 17 nucleotides and their metabolites, 6 amino acids and their metabolites, 5 organic acids and their derivatives, 4 carbohydrates and their metabolites, 3 alcohol and amines, 3 bile acids, 1 FA, 1 benzene, and substituted derivatives were downregulated (Table S2). The upregulated DEMs with an absolute value of Log2FC > 1 between (CK0 and T0)/(CK3 and T3)/(CK7 and T7) included the 13/15/13 amino acid and its metabolites, 1/2/1 nucleotide and its metabolite, 2/2/2 organic acid and its derivatives, 3/8/3 GP, 5/5/5 FA, and 6/6/6 carbohydrates and its metabolites. The downregulated DEMs were the 23/25/21 amino acid and its metabolites, 17/3/5 nucleotide and its metabolite, 10/10/6 organic acid and its derivatives, 7/5/6 GP, 5/2/2 FA, and 3/3/3 carbohydrates and its metabolites. Overall, our findings suggested that the preservative inhibited metabolites production, especially amino acid and its metabolites, nucleotide and its metabolite, and organic acid and its derivatives.

Figure 5I–L show the differential metabolic pathway of eel muscle between CK0 and CK7, and between the CK and T groups under a different storage day. Compared to the CK0 group, the significant differential metabolic pathways found for the CK7 group were ABC transportation, nucleotide metabolism, central carbon metabolism in cancer, glycerophospholipid metabolism, citrate cycle (TCA cycle), biosynthesis of amino acids, carbon metabolism, phospholipase D signaling pathway, etc. (Figure 5I). The significant differential metabolic pathways between the CK0 group and the T0 group were glyoxylate and dicarboxylate metabolism, a glucagon signaling pathway, citrate cycle (TCA cycle), carbon metabolism, central carbon metabolism in cancer, purine metabolism, amino acid metabolism, etc. (Figure 5J). Compared to the CK3 group, the significant differential metabolic pathways in the T3 group were glyoxylate and dicarboxylate metabolism, a glucagon signaling pathway, citrate cycle (TCA cycle), carbon metabolism, central carbon metabolism in cancer, purine metabolism, amino acid metabolism, fructose and manmose metabolism, inositol phosphate metabolism, etc. (Figure 5K). Regarding the CK7 and T7 groups, the differential metabolic pathway was found to be the carbon metabolism, the central carbon metabolism in cancer, amino acid metabolism, nitrogen metabolism, protein digestion and absorption, fructose and mannose metabolism, phosphonate and phosphinate metabolism, and carbon metabolism; these were the main components. Amino acid metabolism and carbon metabolism were the significant differential metabolic pathways in all of the above comparison groups (Figure 5L).

#### 3.5.2. Metabolomics Biomarkers

**Figure 6:**
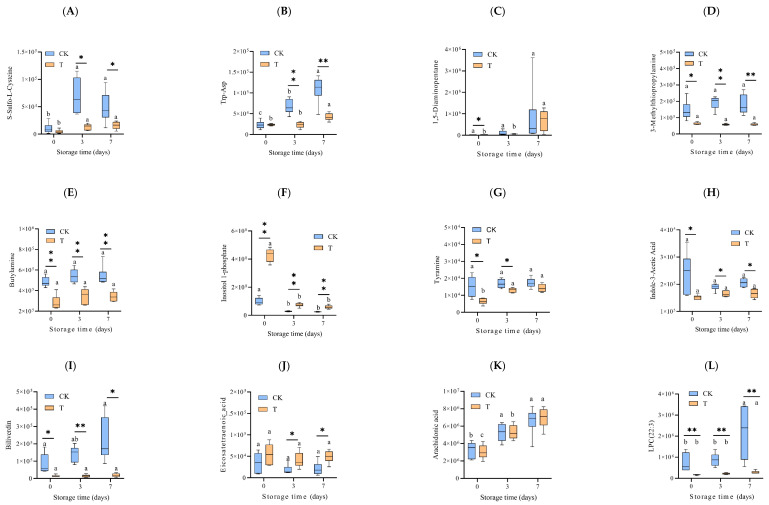

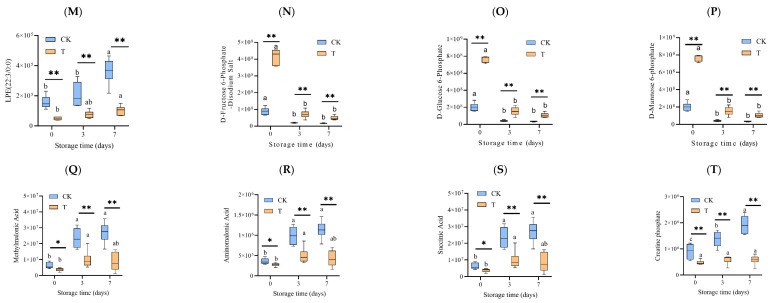
Changes in the relative abundance (mAU/min) of key differential metabolites, which are potentially associated with muscle quality during refrigeration. The differential metabolites were screened out from the LC-MS/MS data with both the VIP value and absolute value of Log2FC > 1. (**A**) S-Sulfo-L-Cysteine, (**B**) Trp-Asp, (**C**) 1,5-Diaminopentane, (**D**) 3-Methylthiopropylamine, (**E**) Butylamine, (**F**) Inositol 1-phosphate, (**G**) Tyramine, (**H**) Indole-3-Acetic Acid, (**I**) Biliverdin, (**J**) Eicosatetraenoic acid, (**K**) arachidonic acid, (**L**) LPC(22:3), (**M**) LPE(22:3/0:0), (**N**) D-Fructose 6-Phosphate-Disodium Salt, (**O**) D-Glucose 6-Phosphate, (**P**) D-Mannose 6-phosphate, (**Q**) Methylmalonic Acid, (**R**) Aminomalonic Acid, (**S**) Succinic Acid, (**T**) Creatine phosphate. CK—control group, T—treatment group with the addition of ε-polylysine and antioxidants; the numbers indicate storage day. Different lowercase letters indicate significant differences (*p* < 0.05) among samples with different storage times. ns indicates no significance, * indicates *p* < 0.05, ** indicates *p* < 0.01.

Among the DEM in the Tables S2–S5 and 20 metabolites were selected as the candidate biomarkers among six groups (CK0, CK3, CK7, T0, T3, and T7), which are potentially related to the quality change. Regardless of the preservation treatment, two amino acids and their metabolites, including S-Sulfo-L-Cysteine and Trp-Asp, increased with the storage time overall (*p* < 0.05) (Figure 6A,B). Compared to the CK0 group, the Log2FC of S-Sulfo-L-Cysteine and Trp-Asp in the CK7 was 2.25 and 2.22, respectively. During storage, the relative content of these substance was significantly lower in the T groups (*p* < 0.05). The Log2FC changes (T versus CK) of S-Sulfo-L-Cysteine were −2.38 and −1.62 at day 3 and day 7, and −1.54 and −1.37 for Trp-Asp, respectively. Among the alcohol and amines, 1, 5-Diaminopentane, 3-Methylthiopropylamine, and Butylamine increased with the storage time (Figure 6C–E), while Inositol 1-phosphate decreased (Figure 6F). Under the same storage time, 1, 5-Diaminopentane and Inositol 1-phosphate in T groups were higher than those in the CK groups, while 3-Methylthiopropylamine and Butylamine were lower (*p* < 0.01).

Tyramine and Indole-3-Acetic Acid belong to Benzene and substituted derivatives and Heterocyclic compounds, respectively. Like the two amino acids and their metabolites, mentioned above, their content increased with the storage time, with the content of T groups being lower than that of the CK group (Figure 6G,H). Biliverdin, one kind of tryptamines, cholines, and pigments, is highly related to the color of eel slices. The Biliverdin of the CK7 sample was unregulated to 1.41 Log2FC of the CK0 sample (Figure 6I). During storage, the Biliverdin of the T3 and T7 samples was dramatically downregulated to −3.26 and −3.56 Log2FC of the CK3 and CK7 samples at day 3 and day 7, respectively.

As for the FA and GP (Figure 6J–M), 12-HETE showed a decreasing tendency, while a significant increasing tendency was found in AA, LPC (22:3), and LPE (22:3/0:0). During storage, the content of 12-HETE was higher in the T group (*p* < 0.05), and the contents of LPC (22:3) and LPE (22:3/0:0) were lower (*p* < 0.01).

Changes in carbohydrates and its metabolites (D-Fructose 6-Phosphate-Disodium Salt, D-Glucose 6-Phosphate, D-Mannose 6-phosphate) and organic acid and its derivatives (Methylmalonic Acid, Aminomalonic Acid, Succinic Acid) are depicted in Figure 6. N-S. The carbohydrates and its metabolites decreased during storage. On the contrary, the organic acid and its derivatives increased. After being in storage for 7 days, the D-Fructose 6-Phosphate-Disodium Salt, D-Glucose 6-Phosphate, and D-Mannose 6-phosphate of the T group were upregulated to 1.56, 1.67, and 1.67 Log2FC for the CK group, while Methylmalonic Acid, Aminomalonic Acid, and Succinic Acid were downregulated to −1.67, −1.42, and −1.67 Log2FC, respectively. Creatine phosphate belongs to nucleotide and its metabolites. Its change tendency is similar to that of the organic acid and its derivatives (Figure 6T).

### 3.6. Correlations Analysis

**Figure 7:**
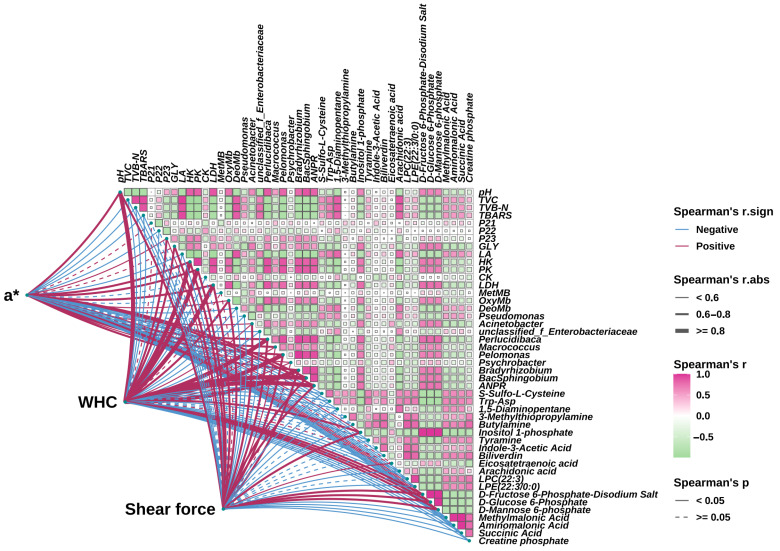
Correlation diagram of key quality indicators, biochemical indicators, bacterial composition, and key metabolites. ANPR: Allorhizobium-Neorhizobium-Pararhizobium-Rhizobium.

A correlation between muscle quality, water status, biochemical indicators, metabolites, and microorganisms of the eel slice muscle samples stored for different times are shown in Figure 7. A significant association among those indices was observed (*p* < 0.05). Furthermore, correlations between key parameters of the eel slice muscle, including a*, WHC, shear force, and other indices, are illustrated by lines of different forms. In detail, a* was positively correlated (0.6 < r < 0.8) with pH, HK, PK, LDH, Perlucidibaca, Macrococcus, Pelomonas, Bradyrhizobium, BacSphingobium, and ANPR (Allorhizobium-Neorhizobium-Pararhizobium-Rhizobium). In addition to these indices, a* was also positively correlated (r < 0.6) with P23, OxyMb, Acinetobacter, Inositol 1-phosphate, D-Fructose 6-Phosphate-Disodium Salt, D-Glucose 6-Phosphate, and D-Mannose 6-phosphate. However, a* had a negative correlation with TVC, TVB-N, TBARS, P22, LA, DetMB, Psychrobacter, S-Sulfo-L-Cysteine, Trp-Asp, 1,5-Diaminopentane, Tyramine, Biliverdin, Arachidonic acid, LPC (22:3), LPE (22:3/0:0), Methylmalonic Acid, Aminomalonic Acid, Succinic Acid, and Creatine phosphate. The correlations of both WHC and shear force with other indices were much similar to that of a*. The differences included that WHC exhibited a higher correlation (r ≥ 0.8) with pH than a*. In addition, shear was not correlated (*p* ≥ 0.05) with Biliverdin or LPC (22:3).

## 4. Discussion

### 4.1. The Deterioration of Slice Quality During Refrigeration

During the refrigeration storage at 4 °C, the quality of eels is prone to deterioration. Based on previous extensive research, it has been determined that this is mainly related to their own metabolism and the action of microorganisms [5,24,25]. We found that muscle quality indicators such as a*, WHC, Shear force, and pH gradually decreased during storage, while TVC, TVB-N, and TBARS gradually increased, which is consistent with the results reported by Özogul et al. [26] and Yanar et al. [27] on European eel (*Anguilla anguilla*). Among the above indicators, a*, WHC, and Shear force directly affect the quality of food consumption and determine its commercial value.

Antibacterial and antioxidant agents are commonly used to maintain the quality of fish products during refrigeration storage [28]. ε-PL exerts antibacterial effects by acting on the cell wall and membrane system, genetic material or genetic particle structure, as well as enzymes or functional proteins [25]. The two enol hydroxyl groups on the 2nd and 3rd carbon atoms of the lactone ring structure of Vc are easily dissociated to release H^+^, which enables Vc to play an important antioxidant role. EGCG is a polyphenolic compound that can exert antioxidant effects through various mechanisms, including the direct scavenging of free radicals, metal chelation, and enhancing the activity of other antioxidants. Pht is a dihydrochalcone compound, belonging to the flavonoid class, with a wide range of sources and strong antioxidant activity. The mixture we added contains ε -PL, VC, EGCG, and Pht, which have synergistic antibacterial and antioxidant effects. Therefore, after this addition, the quality of eel slices was effectively improved.

#### 4.1.1. Change in a*

Color is the most important edible quality of eels. High-quality eels are bright red in color, while low-quality eels are brown in color. Compared to other color indices, the change in a* was most significant during storage (Table 1), indicating that the fade in red color is the main color change, which is consistent with the visual observation (Figure S1B,C). The main reason for the decrease in a* is related to the transition of the myoglobin form (Figure S2). Myoglobin exists in three forms, including deoxymyoglobin (DeoMb), oxymyoglobin (OxyMb), and metmyoglobin (MetMb), which are purple red, bright red, and brown in color, respectively. The iron in DeoMb’s heme is in the ferrous state. Low oxygen partial pressure can maintain myoglobin (Mb) in a deoxygenated state. The iron in OxyMb formed by the combination of Mb and O_2_ remain in the ferrous state, but the stability of Mb has changed. After the oxidation reaction, the Fe^2+^ of hemoglobin in Mb is oxidized to Fe^3+^, and the bound oxygen atoms are released. DeoMb is reduced to MetMb, causing the fish meat to appear brownish in color [29].

pH value, microbial growth, and oxidative active substances can all affect the state of myoglobin [29]. When the pH value is around neutral, the spatial structure of myoglobin is more compact, the chemical properties are more stable, and it is less prone to redox reactions, which is beneficial for maintaining the stability of the meat color. When the pH value decreases, the rate of oxidation of OxyMb to MetMb increases, resulting in a deterioration of the appearance color of the meat [30]. Microorganisms are also one of the main factors leading to the deterioration of meat color. The activity of aerobic bacteria can cause a decrease in oxygen partial pressure on the surface of meat, which is beneficial for the generation and accumulation of MetMb and can lead to changes in meat color [29]. After the death of fish, the fat is unstable and prone to fat degradation, producing some secondary metabolites. The substances produced by degradation can further generate active free radicals or small molecules, such as aldehydes and ketones. Firstly, they can react with Mb to accelerate the generation of MetMb. Secondly, these substances can further generate active free radicals or small molecules, such as aldehydes and ketones, disrupting the normal electron transfer in the mitochondrial respiratory chain and reducing the efficiency of the MetMb reduction system [31]. Compared to CK, the relative content of OxyMb in group T was higher and the relative content of MetMb was lower during storage (Figure S2). This is because the composite preservative in group T increased the pH of the muscle (Figure 1C), reduced the generation of oxidation products (Figure 1F), and inhibited microbial growth (Figure 1D). As a result, a* significantly improved.

We found that biliverdin significantly increased during storage (Figure 6I), and the change in a* was significantly negatively correlated with biliverdin (Figure 7). Biliverdin is a dark green pigment produced by the normal metabolism of hemoglobin. After adding a composite preservative, the content of biliverdin significantly decreased. Therefore, preservative treatment can increase a*, which may be related to preventing the oxidation of Mb to MetMb and the degradation of hemoglobin as well.

#### 4.1.2. Change in WHC

Water holding capacity is also an important quality of muscle food, and its changes are related to muscle tissue and cell integrity, pH, moisture content, and moisture status [32]. After slaughter, muscle cells begin to enter the programmed cell death stage. As a result, cell shrinkage, increased intercellular space, and partial cell lysis lead to a decrease in water holding capacity [6]. Apoptosis, as a common and highly regulated mechanism of programmed cell death used to clear non-essential or damaged organelles, plays an important role in maintaining homeostasis and is an important strategy for regulating energy consumption in organisms and tissues to combat stress [33]. Through correlation analysis (Figure 7), we found that WHC was positively correlated with GLY (0.6 < r < 0.8), LA (r < 0.6), HK (r ≥ 0.8), PK (r ≥ 0.8), and CK (r < 0.6). After slaughtering the fish, blood circulation stops, the oxygen supply is insufficient, and glycolysis rapidly breaks down glycogen into lactic acid (Figure 3A,B), resulting in a decrease in pH (Figure 1C). HK is the first rate-limiting enzyme in the process of glycolysis, and after slaughter, glucose in fish is converted to glucose-6-phosphate and accumulated under its action (Figure 6O). When the concentration of glucose-6-phosphate is high, it will have a certain inhibitory effect on HK, resulting in a decrease in HK activity [34]. PK is an important rate-limiting enzyme in glycolysis, which can catalyze the conversion of phosphoenolpyruvate to pyruvate. After slaughter, fish begin to undergo sugar metabolism and ATP degradation in their bodies. The AMP content continuously increases under the action of this enzyme, leading to a continuous decrease in ATP/AMP, thereby activating PK and increasing its activity to its maximum value [35]. The higher the CK activity, the faster the process of glycolysis [36]. During storage, muscle cell energy metabolism is disrupted, manifested as LA accumulation (Figure 3A), and abnormal glucose activity, such as HK, PK, and CK (Figure 3C,D,F). Through metabolomics analysis, we also found that most enriched metabolic pathways are related to energy metabolism, such as the glucose signaling pathway, citrate cycle (TCA cycle), carbon metabolism, purine metabolism, amino acid metabolism, fructose and mannose metabolism, nitrogen metabolism, protein digestion and absorption, etc. (Figure 6I–L). The composite preservative may delay programmed cell death and increase WHC by maintaining normal energy metabolism.

However, microbial activity during storage may concurrently influence these processes. WHC is positively correlated with pH (r ≥ 0.8) (Figure 7). As glycogen gradually converts into lactate, the pH gradually decreases and approaches the isoelectric point of myofibrillar proteins, leading to a decrease in WHC [37]. During storage, water evaporated, and thus the surface of the eel slices dried, leading to the decreased proportion of free water (Table S1). Under the action of the proteases from microorganisms and the eel slice, protein and carbohydrate compounds with a strong hydrophilic ability were degraded (Table S2), resulting in a decrease in the proportion of bound water (Table S1). The decrease in free water and bound water content led to an increase in the calculated proportion of immobilized water. Generally speaking, a high proportion of immobilized water corresponds to a higher water holding capacity [38], which is contrary to our results. Generally speaking, centrifugation is more sensitive to free water loss, while MRI is more sensitive to bound water status [39,40]. During storage, there is an increase in intercellular space and cell lysis [41], resulting in a significant reduction in the restrictive effect of muscle tissue on water. This leads to a decrease in the resistance of this part of water to centrifugation, making it easier to separate. Therefore, although the proportion of immobilized water increased, the water holding capacity measured by the centrifugation method decreased. The composite preservatives delayed the decrease in pH (Figure 1C), inhibited microbial growth (Figure 1D), and thus enhanced WHC by mitigating both the endogenous biochemical changes (pH drop, apoptosis) and the exogenous microbial contribution (protease activity, spoilage).

#### 4.1.3. Change in Shear Force

The changes in shear force are related to muscle stiffness, muscle cell integrity, and muscle fiber ultrastructure [42]. Compared to 0 days, there was no significant change in shear force after 1 day of refrigeration. Subsequently, the shear force gradually decreased. We did not observe any rigidity phenomenon, indicating that the rigidity period of eels is relatively short. In the early stage of refrigeration, there is an increase in reactive oxygen species, and the activity of enzymes related to sugar metabolism changes, leading to energy metabolism disorders and programmed cell death of muscle cells. During this process, the intercellular space becomes larger and lyses [6]. The simultaneous secretion of Caspase series enzymes leads to the destruction of muscle fiber ultrastructure. During the middle stage of refrigeration, cells begin to self-dissolve. The proteases B, D, and L, secreted in lysosomes, further hydrolyze myofibrillar proteins [3]. In the later stage of refrigeration, microorganisms such as *Pseudomonas* grow rapidly (Figure 4B) and produce a large amount of enzymes that hydrolyze proteins. Therefore, we observed a gradual increase in TVB-N content (Figure 1E), enrichment of metabolic pathways related to protein breakdown (Figure 5I–L), and significant upregulation of amino acids and their metabolites (Table S2). The antioxidants in the composite preservative inhibited the generation of signaling molecules such as ROS, thereby delaying the cell death process [6]. On the other hand, the ε-PL in the composite preservative inhibited microbial growth. Therefore, the shear force significantly increased. Therefore, the change in shear force is not only attributed to the biochemical process, but the role of microorganisms cannot be ignored either.

### 4.2. Changes in Microorganisms During Refrigeration Storage

During the refrigeration storage, the diversity and richness of microorganisms gradually decreased (Figure 4D–F), with *Pseudomonas* becoming the dominant microorganisms (Figure 4B), which is the result of microbial succession in low-temperature environments [43,44]. *Pseudomonas* is a typical low-temperature spoilage bacterium that is directly related to fish spoilage and has strong protein and fat degrading abilities [43]. It was negatively correlated with a*, WHC, and shear force (Figure 7). In this study, *Pseudomonas* was also significantly correlated with GLY, S-Sulfo-L-Cysteine, Trp-Asp, 1, 5-Diaminopentane, Tyramine, D-Glucose 6-Phosphate, D-Mannose 6-phosphate, Succinic Acid, and other metabolites, suggesting its potential to influence the metabolism of eel slices and serve as an important microbiome biomarker of spoilage in refrigeration storage. However, the association of the other increased genera with meat quality has yet to be confirmed. The addition of a composite preservative significantly reduced the proportion of *Psychromonas*. Jia et al. [44] and Liu et al. [45] also found that ε-PL effectively inhibited the growth of *Psychromonas* in Pacific white shrimp (*Litopenaeus vannamei*) and bighead carp (*Aristichthys nobilis*) fillets under refrigeration storage, respectively. The inhibition of *Psychromonas* by ε-PL may be related to the disruption of the cell wall and membrane system, genetic material or genetic particle structure, as well as the denaturation of enzymes or functional proteins [25]. Through LEfSe analysis (Figure 4H,I), we identified microorganisms that have a significant impact on microbial composition differences, including *Bacteroidia* and *Bacteroidota*, which can serve as important biomarkers for microbial composition changes.

### 4.3. Changes in Metabolites During Refrigeration Storage

Under the action of microorganisms and the metabolism of fish meat itself, macromolecules like fish protein and carbohydrates are gradually degraded into low molecular weight metabolites such as amino acids, monosaccharides, and ammonia [9]. Therefore, the glucose signaling pathway, citrate cycle (TCA cycle), carbon metabolism, purify metabolism, amino acid metabolism, fructose and mannose metabolism, nitrogen metabolism, protein digestion, and absorption pathways were significantly enriched in our study (Figure 5). Previous studies have confirmed that these metabolic pathways are closely related to meat spoilage [15]. The accumulation of these metabolites initiates the decay process of meat, leading to discoloration, decreased WHC, decreased shear force, and the production of odors. The addition of a composite preservative inhibited the metabolic processes of eel slices and microorganisms, thereby altering the morphology of metabolites.

By applying statistical methods, we selected 20 DEMs as candidate biomarkers. Among these candidate biomarkers, the relationship between some substances and quality deterioration has been extensively documented. For example, 1,5-Diaminopentane and Tyramine are produced by protein degradation during the decay process, among which 1,5-Diaminopentane has a putrid odor [46]. Biliverdin is produced by the degradation of myoglobin and can affect the color of fish meat. Arachidonic acid plays an important role in the oxidation of fatty acids [47]. The most commonly screened biomarkers include substances related to energy metabolism, such as D-Glucose 6-phosphate D-Mannose 6-phosphate, Succinic Acid, and Creatine phosphate. Glucose-6-phosphate is a common intermediate in various sugar metabolism pathways, which connects numerous pathways of sugar catabolism and synthesis metabolism. Therefore, glucose-6-phosphate is the intersection of various sugar metabolism pathways [34]. The metabolic process of D-mannose involves multiple key enzymes and metabolic pathways, which play an important role in energy supply and biosynthesis. Succinic acid is an intermediate metabolite in the citric acid cyc [34]. Phosphocreatine is an essential high-energy phosphate compound present in muscles for energy metabolism. Energy metabolism can affect cellular state, which in turn affects WHC and shear force [33]. Changes in these energy metabolites are primarily driven by post-mortem muscle biochemistry, but their levels can be modulated by microbial consumption or production during storage. In addition to the substances mentioned above, S-Sulfo-L-Cysteine, Trp-Asp, Inositol 1-phosphate, LPC (22:3), LPE (22:3/0:0), Methylmalonic Acid, and Aminomalonic Acid are also significantly correlated with muscle quality indices (a*, WHC, shear force) (Figure 7), indicating that they can also serve as important biomarkers for the quality changes of eel slices during refrigeration. However, the mechanism of their association with muscle quality still needs further research, including delineating the relative contributions of muscle vs. microbial metabolism to their accumulation.

## 5. Conclusions

This study demonstrates the efficacy of a composite preservative (ε-polylysine, Vc, EGCG, phloretin) for fresh eel slices, but several limitations should be considered. Biological variability in eel physiology, such as size, nutritional status, and pre-slaughter stress, could affect metabolite levels and post-mortem biochemical changes, suggesting that larger sample sizes would help mitigate these factors. While the results are specific to fresh eel, they may not directly apply to other species with different biochemical compositions, although the preservative mechanism could offer promising applications for preserving other high-fat or protein-rich aquatic products. This study provides a foundation for further research into the preservation of aquatic products. Future works could focus on exploring the broader applicability of the composite preservative in preserving other species, as well as investigating its potential in different storage conditions. By enhancing product stability and maintaining quality during storage, this preservative could offer valuable benefits in reducing spoilage and extending market availability.

## Figures and Tables

**Table 1:** Changes in color of rice-field eel slices during refrigeration storage, as affected by the addition of ε-polylysine and antioxidants.

Groups	Storage Time (Days)	L*	a*	b*	C*	H*	R630/580
	0	39.56 ± 2.30 ^Aa^	2.77 ± 0.42 ^Abc^	8.54 ± 0.67 ^Aa^	8.99 ± 0.60 ^Aa^	71.96 ± 3.25 ^Aa^	1.80 ± 0.04 ^Ac^
	1	40.25 ± 2.58 ^Ab^	3.08 ± 0.57 ^Abc^	8.85 ± 0.78 ^Aa^	9.41 ± 0.54 ^Ab^	71.46 ± 2.31 ^Aa^	1.67 ± 0.09 ^Aab^
CK	3	40.70 ± 1.58 ^Ab^	2.68 ± 0.77 ^Ab^	12.57 ± 0.74 ^Ac^	10.96 ± 0.91 ^Ac^	72.94 ± 4.03 ^Ab^	1.61 ± 0.12 ^Aa^
	5	40.45 ± 1.10 ^Ab^	2.31 ± 0.20 ^Ab^	9.64 ± 2.21 ^Ab^	9.79 ± 2.31 ^Ab^	71.75 ± 1.70 ^Aa^	1.63 ± 0.12 ^Aa^
	7	40.53 ± 1.55 ^Ab^	1.25 ± 0.12 ^Aa^	9.44 ± 1.24 ^Ab^	9.58 ± 1.22 ^Ab^	71.71 ± 0.19 ^Aa^	1.59 ± 0.03 ^Aa^
	0	38.37 ± 2.18 ^Aa^	3.11 ± 0.01 ^Ab^	10.32 ± 0.20 ^Ba^	10.32 ± 0.32 ^Ba^	73.16 ± 0.14 ^Aa^	2.02 ± 0.21 ^Ac^
	1	41.60 ± 2.23 ^Ab^	2.62 ± 0.28 ^Abc^	11.04 ± 0.92 ^Bb^	11.74 ± 1.25 ^Bbc^	76.19 ± 0.94 ^Bb^	1.90 ± 0.19 ^Bb^
TG	3	44.01 ± 3.28 ^Bd^	3.19 ± 0.43 ^Ab^	12.62 ± 1.39 ^Bc^	12.57 ± 0.53 ^Bc^	76.53 ± 1.34 ^Bb^	1.94 ± 0.16 ^Bb^
	5	43.15 ± 2.32 ^Bc^	4.04 ± 0.35 ^Bc^	12.26 ± 0.99 ^Bc^	13.65 ± 0.96 ^Bd^	77.61 ± 0.17 ^Bc^	1.92 ± 0.07 ^Bb^
	7	43.24 ± 0.38 ^Bc^	1.58 ± 0.33 ^Aa^	10.71 ± 0.90 ^Ba^	11.07 ± 0.83 ^Bb^	76.02 ± 2.12 ^Bb^	1.71 ± 0.03 ^Ba^

Note: CK—control group, TG—treatment groups. Different capital letters indicate significant differences among the groups with different storage times (*p* < 0.05). Different lowercase letters indicate significant differences between the CK and TG groups (*p* < 0.05).

## Data Availability

The data presented in this study are available on request from the corresponding author due to the restriction. The data are not publicly available due to privacy or ethical restrictions.

## Supplementary Materials

The following supporting information can be downloaded at: https://www.mdpi.com/article/10.3390/foods14132236/s1, Figure S1. The picture of live rice-field eel (A) and its slice. (B) fresh eel slice, (C) eel slice stored at 4 °C for 7 days. Figure S2. Changes on proportion of myoglobin in different states of rice-field eel slices during refrigeration storage as affected by the addition of ε-polylysine and antioxidants. DeoMb—Deoxymyoglobin, OxyMb—oxymyoglobin, MetMb—metmyoglobin. CK—control group, T—treatment group with the addition of ε-polylysine and antioxidant, the number indicate storage day. Table S1 Changes on the peak area proportions of different water fractions of ricefield eel slices during refrigeration storage as affected by the addition of ε-polylysine and antioxidants. Table S2. Differential metabolite of CK7 vs. CK0 with both VIP and absolute value of Log2FC > 1. Table S3. Differential metabolite of T0 vs. CK0 with both VIP and absolute value of Log2FC > 1. Table S4. Differential metabolite of T3 vs. CK3 with both VIP and absolute value of Log2FC > 1. Table S5. Differential metabolite of T7 vs. CK7 with both VIP and absolute value of Log2FC > 1.
