# Supplementary material for: Integrated Microbiome and Metabolomics Insights into Meat Quality Changes in Rice-Field Eel Slices During Refrigeration Storage: Effects of ε-Polylysine, Vitamin C, Epigallocatechin Gallate, and Phloretin
Source: Foods. 2025 Jun 25;14(13):2236. doi: 10.3390/foods14132236 (PMC12249402; doi:10.3390/foods14132236)
Supplement: Supplementary file 1 [file foods-14-02236-s001.zip › foods-3693317-supplementary.pdf]

Figure S1. The picture of live rice-field eel (A) and its slice. B - fresh eel slice, (C) eel slice stored at 4 ºC for 7 days

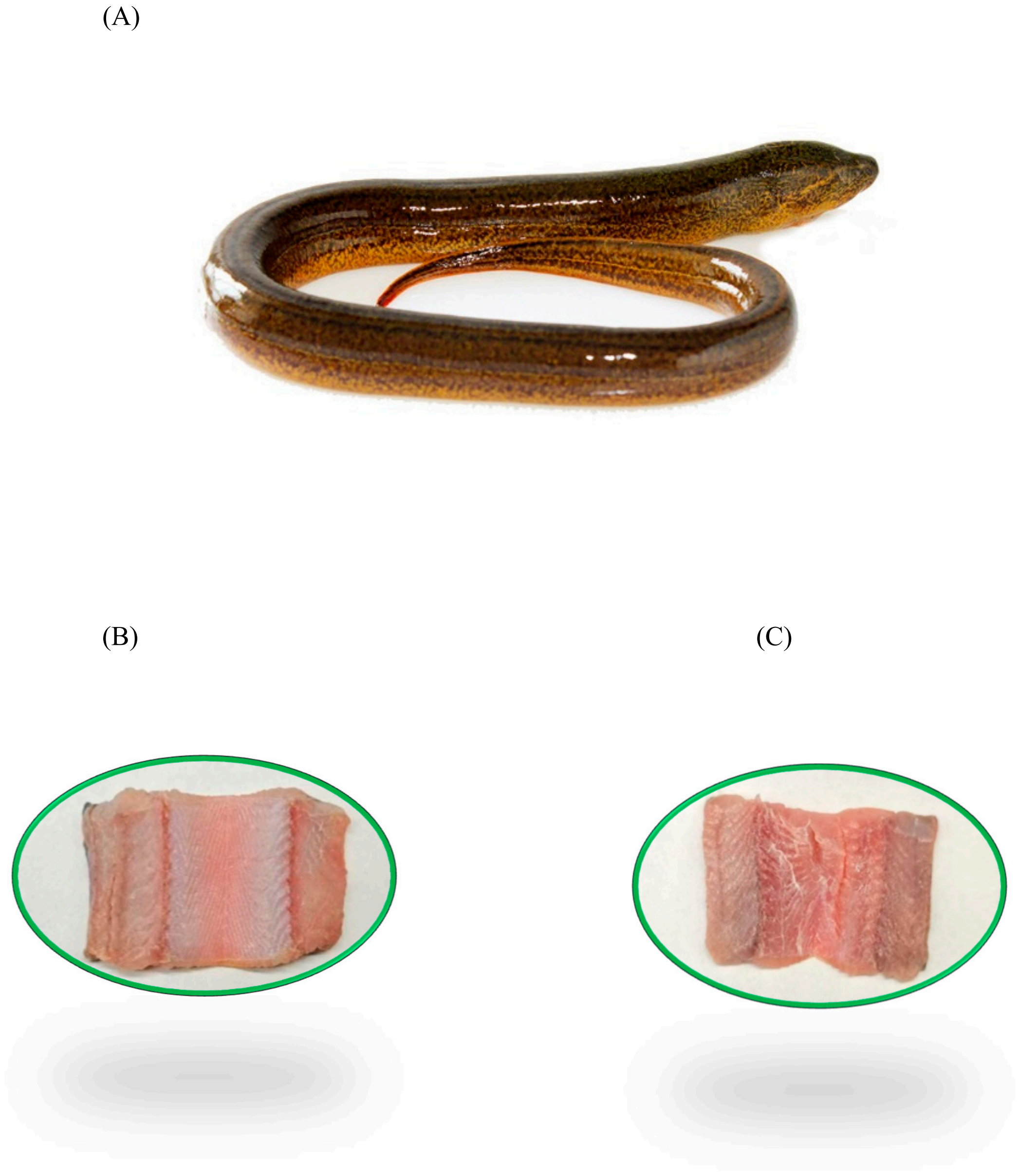

Figure S2. Changes on proportion of myoglobin in different states of rice-field eel slices during refrigeration storage as affected by the addition of ε - polylysine and antioxidants. DeoMb - Deoxymyoglobin, OxyMb - oxymyoglobin, MetMb - metmyoglobin. CK - control group, T - treatment group with the addition of ε - polylysine and antioxidant, the number indicate storage day.

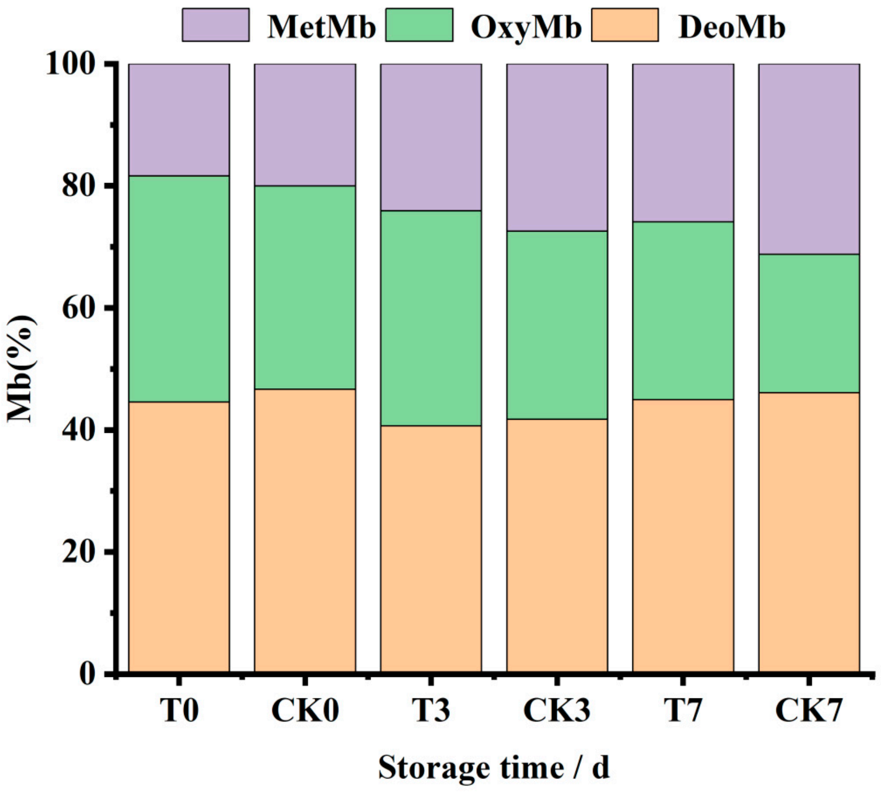

Table S1 Changes on the peak area proportions of different water fractions of rice-field eel slices during refrigeration storage as affected by the addition of ε - polylysine and antioxidants

Groups	Storage time (days)	P_{21} (%)	P_{22} (%)	P_{23} (%)
CK	0	$5.74±0.45^{Aa}$	$90.64±1.16^{Da}$	$3.61±1.38^{Aa}$
	1	$5.58±0.33^{Aa}$	$92.02±0.57^{Ca}$	$2.40±0.24^{ABa}$
	3	$4.72±0.25^{Ba}$	$93.52±0.60^{Ba}$	$1.76±0.44^{Ba}$
	5	$3.34±0.32^{Ca}$	$95.10±0.57^{Aa}$	$1.56±0.83^{Ba}$
	7	$4.40±0.35^{Ba}$	$93.44±0.43^{Ba}$	$2.16±0.19^{Ba}$
TG	0	$3.08±0.56^{Db}$	$94.79±0.88^{ABb}$	$2.13±0.47^{Ab}$
	1	$6.08±0.97^{Aa}$	$93.47±1.00^{Da}$	$1.93±0.09^{Bb}$
	3	$4.99±0.25^{Ba}$	$93.38±0.76C^{a}$	$1.62±0.51^{Aa}$
	5	$4.43±0.02^{BCb}$	$93.69±0.12^{BCb}$	$1.88±0.09^{Aa}$
	7	$3.90±0.22^{CDb}$	$95.42±0.26^{Ab}$	$1.79±0.05^{Bb}$

Note: CK - control group, TG - treatment groups; Different capital letters indicate significant differences among the groups with different storage time ($P<0.05$). Different lowercase letters indicate significant differences between the CK and TG groups ($P<0.05$).

Table S2. Differential metabolite of CK7 vs. CK0 with both VIP and absolute value of Log2FC > 1

	Compounds	Class I	Log2FC	Type
1	Glutathione Oxidized	Amino acid and Its metabolites	5.27	up
2	Thr-Val	Amino acid and Its metabolites	4.72	up
3	Ile-Asn	Amino acid and Its metabolites	4.55	up
4	cyclo(glu-glu)	Amino acid and Its metabolites	4.19	up
5	Pro-Phe	Amino acid and Its metabolites	3.86	up
6	Val-Glu	Amino acid and Its metabolites	3.62	up
7	Proline-Hydroxyproline	Amino acid and Its metabolites	3.40	up
8	Leu-Asp	Amino acid and Its metabolites	3.40	up
9	Gly-Trp	Amino acid and Its metabolites	3.19	up
10	Ile-Ala	Amino acid and Its metabolites	3.07	up
11	Pro-Leu	Amino acid and Its metabolites	3.05	up
12	Glu-Ile	Amino acid and Its metabolites	3.00	up
13	Ile-Glu	Amino acid and Its metabolites	3.00	up
14	Leu-Glu	Amino acid and Its metabolites	3.00	up
15	Thr-Thr	Amino acid and Its metabolites	2.94	up
16	Glu-His	Amino acid and Its metabolites	2.89	up
17	Gly-Ile	Amino acid and Its metabolites	2.87	up
18	Glu-Glu-Ile	Amino acid and Its metabolites	2.82	up
19	Val-His	Amino acid and Its metabolites	2.81	up

20	His-Val	Amino acid and Its metabolites	2.75	up
21	Ala-Glu	Amino acid and Its metabolites	2.66	up
22	Glu-Arg	Amino acid and Its metabolites	2.64	up
23	Asn-Val	Amino acid and Its metabolites	2.58	up
24	Val-Asn	Amino acid and Its metabolites	2.58	up
25	Ala-Asn	Amino acid and Its metabolites	2.57	up
26	Asn-Ala	Amino acid and Its metabolites	2.57	up
27	Glu-Cit	Amino acid and Its metabolites	2.56	up
28	His-Tyr	Amino acid and Its metabolites	2.51	up
29	His-Leu	Amino acid and Its metabolites	2.50	up
30	Gly-Phe	Amino acid and Its metabolites	2.44	up
31	Asn-Leu	Amino acid and Its metabolites	2.39	up
32	Asn-Ile	Amino acid and Its metabolites	2.38	up
33	Ala-gln	Amino acid and Its metabolites	2.37	up
34	His-Ala	Amino acid and Its metabolites	2.36	up
35	Ile-Gln	Amino acid and Its metabolites	2.35	up
36	Ser-Val	Amino acid and Its metabolites	2.33	up
37	Pro-Glu	Amino acid and Its metabolites	2.28	up
38	Tyr-Glu	Amino acid and Its metabolites	2.28	up
39	Ser-Glu	Amino acid and Its metabolites	2.27	up
40	S-Sulfo-L-Cysteine	Amino acid and Its metabolites	2.25	up
41	Oxaceprol	Amino acid and Its metabolites	2.23	up

42	Tyr-Gly	Amino acid and Its metabolites	2.23	up
43	Trp-Asp	Amino acid and Its metabolites	2.22	up
44	Thr-Ile	Amino acid and Its metabolites	2.20	up
45	Thr-Leu	Amino acid and Its metabolites	2.20	up
46	Val-Arg	Amino acid and Its metabolites	2.16	up
47	Leu-Ala	Amino acid and Its metabolites	2.14	up
48	Tyr-Asp	Amino acid and Its metabolites	2.14	up
49	Tyr-Asn	Amino acid and Its metabolites	2.13	up
50	Ser-Arg	Amino acid and Its metabolites	2.12	up
51	Asn-Tyr	Amino acid and Its metabolites	2.11	up
52	Asn-Arg	Amino acid and Its metabolites	2.08	up
53	Ser-Trp	Amino acid and Its metabolites	2.06	up
54	Gly-Tyr	Amino acid and Its metabolites	2.05	up
55	Arg-Glu	Amino acid and Its metabolites	2.03	up
56	Asn-Asp	Amino acid and Its metabolites	2.03	up
57	Gly-Leu	Amino acid and Its metabolites	2.01	up
58	Val-Ile	Amino acid and Its metabolites	2.00	up
59	Val-Leu	Amino acid and Its metabolites	2.00	up
60	Pro-Trp	Amino acid and Its metabolites	1.99	up
61	Asp-Lys	Amino acid and Its metabolites	1.98	up
62	Lys-Asp	Amino acid and Its metabolites	1.98	up
63	Pro-Gln	Amino acid and Its metabolites	1.98	up

64	Val-Gly	Amino acid and Its metabolites	1.98	up
65	Val-Tyr	Amino acid and Its metabolites	1.95	up
66	L-Saccharopine	Amino acid and Its metabolites	1.95	up
67	Val-Trp	Amino acid and Its metabolites	1.90	up
68	Ser-Gln	Amino acid and Its metabolites	1.87	up
69	Ala-His	Amino acid and Its metabolites	1.86	up
70	Val-Gln	Amino acid and Its metabolites	1.85	up
71	L-Carnosine	Amino acid and Its metabolites	1.84	up
72	Phe-His	Amino acid and Its metabolites	1.82	up
73	Ala-Ile	Amino acid and Its metabolites	1.82	up
74	Pro-Arg	Amino acid and Its metabolites	1.80	up
75	Ile-Gly	Amino acid and Its metabolites	1.79	up
76	Ile-Ser	Amino acid and Its metabolites	1.78	up
77	Gly-Thr	Amino acid and Its metabolites	1.76	up
78	Lys-Ser	Amino acid and Its metabolites	1.76	up
79	Thr-His	Amino acid and Its metabolites	1.74	up
80	Arg-Thr	Amino acid and Its metabolites	1.72	up
81	Ile-Val	Amino acid and Its metabolites	1.70	up
82	Gly-Gly	Amino acid and Its metabolites	1.69	up
83	Ala-Ser	Amino acid and Its metabolites	1.68	up
84	Asp-Val	Amino acid and Its metabolites	1.68	up
85	Val-Asp	Amino acid and Its metabolites	1.68	up

86	L-Citrulline	Amino acid and Its metabolites	1.68	up
87	Arg-Ser	Amino acid and Its metabolites	1.67	up
88	Gln-Gln	Amino acid and Its metabolites	1.65	up
89	Asn-Gly	Amino acid and Its metabolites	1.61	up
90	Pro-Met	Amino acid and Its metabolites	1.61	up
91	Ser-Leu	Amino acid and Its metabolites	1.61	up
92	Ser-Ala	Amino acid and Its metabolites	1.60	up
93	Thr-Glu	Amino acid and Its metabolites	1.60	up
94	Pyr-Glu	Amino acid and Its metabolites	1.60	up
95	Leu-Val	Amino acid and Its metabolites	1.59	up
96	His-Trp	Amino acid and Its metabolites	1.56	up
97	Ala-Ala	Amino acid and Its metabolites	1.55	up
98	Pro-Lys	Amino acid and Its metabolites	1.53	up
99	Lys-Thr	Amino acid and Its metabolites	1.51	up
100	3-Hydroxy-L-phenylalanine	Amino acid and Its metabolites	1.51	up
101	Leu-Gly	Amino acid and Its metabolites	1.50	up
102	Ureidosuccinic acid	Amino acid and Its metabolites	1.50	up
103	Asn-Glu	Amino acid and Its metabolites	1.49	up
104	Ser-Ser	Amino acid and Its metabolites	1.47	up
105	N-Acetyl-L-alanine	Amino acid and Its metabolites	1.46	up
106	Thr-Tyr	Amino acid and Its metabolites	1.46	up
107	L-Tyrosine	Amino acid and Its metabolites	1.46	up

108	Lys-Pro	Amino acid and Its metabolites	1.45	up
109	Lys-Met	Amino acid and Its metabolites	1.45	up
110	L-Cystine	Amino acid and Its metabolites	1.44	up
111	Glu-Gln	Amino acid and Its metabolites	1.44	up
112	γ-Glu-Gln	Amino acid and Its metabolites	1.44	up
113	Arg-Ile	Amino acid and Its metabolites	1.44	up
114	Asn-Phe	Amino acid and Its metabolites	1.43	up
115	Phe-Ala-Ser	Amino acid and Its metabolites	1.43	up
116	Ser-Phe-Ala	Amino acid and Its metabolites	1.43	up
117	Leu-Asn	Amino acid and Its metabolites	1.40	up
118	Arg-Ala	Amino acid and Its metabolites	1.34	up
119	Pyroglutamic acid	Amino acid and Its metabolites	1.32	up
120	Leu-Ile	Amino acid and Its metabolites	1.31	up
121	Ala-Arg	Amino acid and Its metabolites	1.31	up
122	Lys-Phe	Amino acid and Its metabolites	1.30	up
123	Glu-Phe	Amino acid and Its metabolites	1.30	up
124	Ser-Phe	Amino acid and Its metabolites	1.29	up
125	cyclo(gly-glu)	Amino acid and Its metabolites	1.28	up
126	N-Acetylglycine	Amino acid and Its metabolites	1.24	up
127	Glu-Ser	Amino acid and Its metabolites	1.22	up
128	Phe-Ser	Amino acid and Its metabolites	1.21	up
129	Ile-Ile	Amino acid and Its metabolites	1.20	up

130	Asp-Glu	Amino acid and Its metabolites	1.20	up
131	Ile-Thr	Amino acid and Its metabolites	1.19	up
132	Leu-Thr	Amino acid and Its metabolites	1.19	up
133	N-Acetylneuraminic Acid(SA)	Amino acid and Its metabolites	1.18	up
134	Ile-Leu	Amino acid and Its metabolites	1.17	up
135	Leu-Leu	Amino acid and Its metabolites	1.17	up
136	Trp-Pro	Amino acid and Its metabolites	1.15	up
137	Ile-Lys	Amino acid and Its metabolites	1.15	up
138	Met-Gln	Amino acid and Its metabolites	1.14	up
139	Asp-Arg	Amino acid and Its metabolites	1.11	up
140	Lys-Leu	Amino acid and Its metabolites	1.06	up
141	N-Acetylthreonine	Amino acid and Its metabolites	(6.04)	down
142	Cysteine-glutathione disulfide	Amino acid and Its metabolites	(2.58)	down
143	Aspartic Acid	Amino acid and Its metabolites	(2.39)	down
144	Glu-Leu	Amino acid and Its metabolites	(1.77)	down
145	S-Adenosyl-L-Methionine	Amino acid and Its metabolites	(1.36)	down
146	4-acetoxyphenol	Benzene and substituted derivatives	4.14	up
147	Dipyrocetyl	Benzene and substituted derivatives	(2.58)	down
148	Hypotaurocyamine	Alcohol and amines	2.35	up
149	N-Acetylhistamine	Alcohol and amines	2.03	up
150	2-(α-D-mannosyl)-3-phosphate glyceride	Alcohol and amines	(4.08)	down
151	Inositol 1-phosphate	Alcohol and amines	(1.98)	down
152	Cholic acid	Bile acids	2.26	up
153	Gamma-Mercholic Acid	Bile acids	2.26	up

154	Deoxycholic acid	Bile acids	2.20	up
155	Tauroursodeoxycholic acid	Bile acids	(2.07)	down
156	LPC(22:3)	GP	1.58	up
157	LPC(17:2/0:0)	GP	1.22	up
158	LPE(22:3/0:0)	GP	1.21	up
159	LPA(18:3)	GP	1.20	up
160	LPC(24:6e)	GP	1.19	up
161	LPE(20:1/0:0)	GP	1.14	up
162	LPE(18:1/0:0)	GP	1.12	up
163	LPA(20:4)	GP	1.10	up
164	LPE(20:2/0:0)	GP	1.10	up
165	LPG(22:5)	GP	1.07	up
166	LPC(18:3/0:0)	GP	1.04	up
167	PC(12:0/12:0)	GP	1.03	up
168	LPE(16:1/0:0)	GP	1.01	up
169	LPC(16:1/0:0)	GP	1.00	up
170	Adenosine 5'-triphosphate（ATP）	Nucleotide and Its metabolites	8.55	up
171	Uridine	Nucleotide and Its metabolites	4.37	up
172	Xanthine	Nucleotide and Its metabolites	2.94	up
173	Uracil	Nucleotide and Its metabolites	2.90	up
174	Guanosine	Nucleotide and Its metabolites	2.79	up
175	Oxypurinol	Nucleotide and Its metabolites	2.42	up
176	Xanthosine	Nucleotide and Its metabolites	1.64	up
177	Cytidine	Nucleotide and Its metabolites	1.53	up
178	Isocytosine	Nucleotide and Its metabolites	1.27	up
179	2-Methylguanosine	Nucleotide and Its metabolites	1.26	up
180	1-Methyladenosine	Nucleotide and Its metabolites	1.17	up

181	N6-methyladenosine	Nucleotide and Its metabolites	1.17	up
182	Creatine phosphate	Nucleotide and Its metabolites	1.09	up
183	Cytidine 2',3'-Cyclic Monophosphoric Acid	Nucleotide and Its metabolites	1.08	up
184	2-Hydroxy-6-Aminopurine	Nucleotide and Its metabolites	1.04	up
185	Guanine	Nucleotide and Its metabolites	1.04	up
186	Inosine	Nucleotide and Its metabolites	1.00	up
187	Inosine diphosphate	Nucleotide and Its metabolites	(4.64)	down
188	Inosine 5'-monophosphate	Nucleotide and Its metabolites	(4.56)	down
189	Guanosine-5'-monophosphate	Nucleotide and Its metabolites	(3.79)	down
190	Uridine 5’-Diphosphate	Nucleotide and Its metabolites	(3.76)	down
191	Uridine-5'-diphospho-N-acetylgalactosamine disodium salt	Nucleotide and Its metabolites	(3.37)	down
192	Guanosine-5'-diphosphate	Nucleotide and Its metabolites	(2.77)	down
193	3'-Adenylic acid	Nucleotide and Its metabolites	(2.69)	down
194	Adenosine 5'-Monophosphate	Nucleotide and Its metabolites	(1.27)	down
195	GDP-L-fucose	Nucleotide and Its metabolites	(1.25)	down
196	Adenosine-5'-Diphosphoglucose	Nucleotide and Its metabolites	(1.20)	down
197	5'-Deoxy-5'-fluoroadenosine	Nucleotide and Its metabolites	(1.17)	down
198	Uridine 5-Monophosphate	Nucleotide and Its metabolites	(1.06)	down
199	Adenosine	Nucleotide and Its metabolites	(1.04)	down
200	Butenoyl-PAF	Others	1.00	up
201	(4Z,7Z,10Z,13Z,16Z,19Z)-Eicosahexaenoate	Aldehyde,Ketones,Esters	1.95	up
202	Biliverdin	Tryptamines,Cholines,Pigments	1.41	up

203	D-Sedoheptuiose 7-Phosphate	Carbohydrates and Its metabolites	2.54	up
204	N-Acetylglucosamine 1-Phosphate	Carbohydrates and Its metabolites	2.34	up
205	2-O-α-D-glucopyranosyl-L-ascorbic acid	Carbohydrates and Its metabolites	1.94	up
206	UDP-glucose	Carbohydrates and Its metabolites	(6.09)	down
207	D-Glucose 6-Phosphate	Carbohydrates and Its metabolites	(2.61)	down
208	D-Mannose 6-phosphate	Carbohydrates and Its metabolites	(2.61)	down
209	D-Fructose 6-Phosphate-Disodium Salt	Carbohydrates and Its metabolites	(2.40)	down
210	Uric acid	Organic acid and Its derivatives	4.22	up
211	Argininosuccinic acid	Organic acid and Its derivatives	2.22	up
212	ST-638	Organic acid and Its derivatives	2.16	up
213	Methylmalonic Acid	Organic acid and Its derivatives	2.14	up
214	Succinic Acid	Organic acid and Its derivatives	2.14	up
215	α-Hydroxyglutaric Acid (sodium salt)	Organic acid and Its derivatives	2.10	up
216	Aminomalonic Acid	Organic acid and Its derivatives	1.66	up
217	2-amino-4-oxovaleric acid	Organic acid and Its derivatives	1.46	up
218	Hydroxyphenyllactic acid	Organic acid and Its derivatives	1.46	up
219	2-Hydroxyglutaric acid	Organic acid and Its derivatives	1.43	up
220	3-Hydroxyglutaric acid	Organic acid and Its derivatives	1.43	up
221	Citramalic Acid	Organic acid and Its derivatives	1.43	up
222	Mevalonate 5-phosphate	Organic acid and Its derivatives	1.41	up
223	Guanidinoethyl Sulfonate	Organic acid and Its derivatives	1.10	up
224	DL-Aminopimelic acid	Organic acid and Its derivatives	(3.27)	down

225	5-O-(1-carboxyvinyl)-3-phosphate	Organic acid and Its derivatives	(2.50)	down
226	Phosphoenolpyruvate	Organic acid and Its derivatives	(2.11)	down
227	Iminodiacetic acid	Organic acid and Its derivatives	(1.92)	down
228	α-Ketoglutaric Acid (α-KG)	Organic acid and Its derivatives	(1.88)	down
229	2,4-Dihydroxypyridine	Heterocyclic compounds	2.83	up
230	1,4-Dihydro-1-Methyl-4-Oxo-3-Pyridinecarboxamide	Heterocyclic compounds	2.43	up
231	1-pyrroline-4-hydroxy-2-carboxylate	Heterocyclic compounds	1.87	up
232	6-Methylnicotinamide	Heterocyclic compounds	1.06	up
233	12,13-EpOME	FA	8.55	up
234	Carnitine C12:1-2OH	FA	1.76	up
235	FFA(22:5)	FA	1.32	up
236	FFA(22:4)	FA	1.18	up
237	FFA(20:2)	FA	1.14	up
238	FFA(22:6)	FA	1.11	up
239	FFA(18:4)	FA	1.10	up
240	AA	FA	1.01	up
241	Carnitine C10:2	FA	(1.81)	down

Note: CK - control group, T - treatment group with the addition of ε - polylysine and antioxidant, the number indicate storage day. The numbers in parentheses are negative values.

Table S3. Differential metabolite of T0 vs. CK0 with both VIP and absolute value of Log2FC > 1

	Compounds	Class I	Log2FC	Type
1	Asn-Gln	Amino acid and Its metabolites	2.07	up
2	Ser-Phe	Amino acid and Its metabolites	1.67	up
3	Phe-Ser	Amino acid and Its metabolites	1.60	up
4	L-Cystathionine	Amino acid and Its metabolites	1.35	up
5	Pro-Met	Amino acid and Its metabolites	1.22	up
6	Ile-Leu	Amino acid and Its metabolites	1.16	up
7	Leu-Leu	Amino acid and Its metabolites	1.16	up
8	Leu-Ile	Amino acid and Its metabolites	1.09	up
9	L-Arginine	Amino acid and Its metabolites	1.05	up
10	Tyr-Ala	Amino acid and Its metabolites	1.05	up
11	N-Alpha-Acetyl-L-Asparagine	Amino acid and Its metabolites	1.04	up
12	Ile-Ile	Amino acid and Its metabolites	1.03	up
13	Glu-Glu-Ile	Amino acid and Its metabolites	1.01	up
14	N-Acetylthreonine	Amino acid and Its metabolites	(10.27)	down
15	Thr-Trp	Amino acid and Its metabolites	(3.49)	down
16	Asn-Pro	Amino acid and Its metabolites	(2.26)	down
17	Pro-Asn	Amino acid and Its metabolites	(2.26)	down
18	Nα-Acetyl-L-Arginine	Amino acid and Its metabolites	(2.20)	down
19	γ-Glu-Cys	Amino acid and Its metabolites	(1.96)	down

20	Cysteine-glutathione disulfide	Amino acid and Its metabolites	(1.96)	down
21	Ile-Trp	Amino acid and Its metabolites	(1.74)	down
22	Lys-Leu	Amino acid and Its metabolites	(1.62)	down
23	1-Methylhistidine	Amino acid and Its metabolites	(1.55)	down
24	Trp-His	Amino acid and Its metabolites	(1.45)	down
25	Ser-Gln	Amino acid and Its metabolites	(1.36)	down
26	Ile-Lys	Amino acid and Its metabolites	(1.34)	down
27	Val-Val	Amino acid and Its metabolites	(1.31)	down
28	L-Theanine	Amino acid and Its metabolites	(1.29)	down
29	Nopaline	Amino acid and Its metabolites	(1.27)	down
30	2-amino-6-oxohexanoic acid	Amino acid and Its metabolites	(1.26)	down
31	L-Glycine	Amino acid and Its metabolites	(1.18)	down
32	S-Allyl-L-cysteine	Amino acid and Its metabolites	(1.17)	down
33	L-Serine	Amino acid and Its metabolites	(1.17)	down
34	L-Cystine	Amino acid and Its metabolites	(1.14)	down
35	γ-Glu-Met	Amino acid and Its metabolites	(1.12)	down
36	N-Amidino-L-Aspartate	Amino acid and Its metabolites	(1.11)	down
37	N-(3-Hydroxypropyl)phthalimide	Benzene and substituted derivatives	(2.70)	down
38	3-Amino-2-naphthoic acid	Benzene and substituted derivatives	(2.68)	down
39	N-(1-naphthyl) carbamoyl benzoic acid	Benzene and substituted derivatives	(1.74)	down
40	Dipyrocetyl	Benzene and substituted derivatives	(1.72)	down
41	4-hydroxybenzylamine	Benzene and substituted derivatives	(1.68)	down

42	4-Methoxysalicylic Acid	Benzene and substituted derivatives	(1.51)	down
43	3,4-Dihydroxybenzeneacetic Acid	Benzene and substituted derivatives	(1.38)	down
44	Tyramine	Benzene and substituted derivatives	(1.25)	down
45	Trolox	Benzene and substituted derivatives	(1.19)	down
46	2-Carboxybenzaldehyde	Benzene and substituted derivatives	(1.14)	down
47	Ellagic acid	Benzene and substituted derivatives	(1.09)	down
48	4-(4-Hydroxyaniline)-6,7-Dimethoxyquinazoline	Benzene and substituted derivatives	(1.04)	down
49	Inositol 1-phosphate	Alcohol and amines	2.13	up
50	4-methyl-5-thiazole-ethanol	Alcohol and amines	1.14	up
51	Bis(1-inositol)-3,1'-phosphate 1-phosphate	Alcohol and amines	1.05	up
52	p-Hydroxyphenylethanolamine	Alcohol and amines	(2.44)	down
53	3-Methylthiopropylamine	Alcohol and amines	(1.20)	down
54	Murideoxycholic acid	Bile acids	(2.16)	down
55	Isochodeoxycholic acid	Bile acids	(2.01)	down
56	3-Epideoxycholic acid	Bile acids	(1.87)	down
57	Tauro-beta-muricholic acid	Bile acids	(1.21)	down
58	Taurohyocholic acid	Bile acids	(1.21)	down
59	Tauroursodeoxycholic acid	Bile acids	(1.18)	down
60	Thiamine	CoEnzyme and vitamins	1.43	up
61	LPC(24:6e)	GP	1.19	up
62	LPC(24:6/0:0)	GP	1.06	up
63	LPC(17:2/0:0)	GP	1.05	up
64	LPC(22:3)	GP	(2.16)	down
65	LPC(O-18:2)	GP	(1.70)	down
66	LPE(0:0/20:2)	GP	(1.67)	down
67	LPE(22:3/0:0)	GP	(1.66)	down
68	LPE(20:2/0:0)	GP	(1.24)	down
69	LPE(0:0/22:4)	GP	(1.04)	down
70	LPC(O-14:0)	GP	(1.00)	down
71	Uridine-5'-diphospho-N-acetylgalactosamine disodium salt	Nucleotide and Its metabolites	1.41	up
72	Guanosine 3',5'-Cyclic Monophosphate	Nucleotide and Its metabolites	(2.28)	down

73	N6-Succinyl Adenosine	Nucleotide and Its metabolites	(1.67)	down
74	2-Aminomethylpyrimidine	Nucleotide and Its metabolites	(1.02)	down
75	Biliverdin	Tryptamines,Cholines,Pigments	(2.52)	down
76	Sucrose 6′-monophosphate	Carbohydrates and Its metabolites	2.62	up
77	D-Fructose 6-Phosphate-Disodium Salt	Carbohydrates and Its metabolites	2.21	up
78	D-Glucose 6-Phosphate	Carbohydrates and Its metabolites	1.92	up
79	D-Mannose 6-phosphate	Carbohydrates and Its metabolites	1.92	up
80	Glucaric acid	Carbohydrates and Its metabolites	1.29	up
81	Mucic Acid	Carbohydrates and Its metabolites	1.29	up
82	2-O-α-D-glucopyranosyl-L-ascorbic acid	Carbohydrates and Its metabolites	(2.86)	down
83	UDP-glucose	Carbohydrates and Its metabolites	(2.51)	down
84	Ribonic Acid	Carbohydrates and Its metabolites	(1.13)	down
85	Uric acid	Organic acid and Its derivatives	1.60	up
86	Phosphonoacetic acid	Organic acid and Its derivatives	1.49	up
87	Hydroxyphenyllactic acid	Organic acid and Its derivatives	1.14	up
88	Hypaphorine	Organic acid and Its derivatives	(4.54)	down
89	Medicagenic acid	Organic acid and Its derivatives	(2.69)	down
90	2-Hydroxy-4-(methylthio)butyric acid	Organic acid and Its derivatives	(2.50)	down
91	Traumatic acid	Organic acid and Its derivatives	(1.95)	down
92	Caffeic Acid	Organic acid and Its derivatives	(1.84)	down
93	2-Amino-3-phosphonopropionic acid	Organic acid and Its derivatives	(1.71)	down
94	2-Aminoethanesulfinic Acid	Organic acid and Its derivatives	(1.52)	down

95	Mevalonate 5-phosphate	Organic acid and Its derivatives	(1.20)	down
96	3-Methylsalicylic acid	Organic acid and Its derivatives	(1.19)	down
97	Guanidineacetic Acid	Organic acid and Its derivatives	(1.14)	down
98	Indoxylsulfuric acid	Heterocyclic compounds	(3.39)	down
99	Methoxyindoleacetic Acid	Heterocyclic compounds	(2.72)	down
100	Indole-3-lactic acid	Heterocyclic compounds	(1.53)	down
101	N'-Methyl-2-pyridone-5-carboxamide	Heterocyclic compounds	(1.20)	down
102	Carnitine C12:1-2OH	FA	2.15	up
103	FFA(18:4)	FA	1.48	up
104	Carnitine C10:2	FA	1.24	up
105	Carnitine C18:4	FA	1.15	up
106	Carnitine ph-C1	FA	1.10	up
107	Carnitine C5:0	FA	(2.55)	down
108	Carnitine-2-methyl-C4	FA	(2.55)	down
109	Carnitine C7:DC	FA	(1.68)	down
110	17(18)-EpETE	FA	(1.64)	down
111	Carnitine C8-OH	FA	(1.12)	down

Note: CK - control group, T - treatment group with the addition of ε - polylysine and antioxidant, the number indicate storage day. The numbers in parentheses are negative values.

Table S4. Differential metabolite of T3 vs. CK3 with both VIP and absolute value of Log2FC > 1

	Compounds	Class I	Log2FC	Type
1	Phe-Met	Amino acid and Its metabolites	1.68	up
2	Ser-Phe	Amino acid and Its metabolites	1.61	up
3	Met-Phe	Amino acid and Its metabolites	1.48	up
4	Phe-Ser	Amino acid and Its metabolites	1.46	up
5	Tyr-Ala	Amino acid and Its metabolites	1.39	up
6	Ala-Tyr	Amino acid and Its metabolites	1.29	up
7	Ile-Leu	Amino acid and Its metabolites	1.25	up
8	Leu-Leu	Amino acid and Its metabolites	1.25	up
9	Ile-Ile	Amino acid and Its metabolites	1.22	up
10	Pro-Met	Amino acid and Its metabolites	1.17	up
11	Leu-Ile	Amino acid and Its metabolites	1.16	up
12	Ala-Val	Amino acid and Its metabolites	1.14	up
13	L-Cystathionine	Amino acid and Its metabolites	1.10	up
14	Val-Ile	Amino acid and Its metabolites	1.08	up
15	Val-Leu	Amino acid and Its metabolites	1.08	up
16	2-amino-6-oxohexanoic acid	Amino acid and Its metabolites	(5.00)	down
17	Nα-Acetyl-L-Arginine	Amino acid and Its metabolites	(3.04)	down
18	L-Theanine	Amino acid and Its metabolites	(2.69)	down
19	S-Sulfo-L-Cysteine	Amino acid and Its metabolites	(2.38)	down
20	γ-Glu-Met	Amino acid and Its metabolites	(2.03)	down
21	γ-Glu-Cys	Amino acid and Its metabolites	(1.92)	down
22	L-Cystine	Amino acid and Its metabolites	(1.82)	down
23	Thr-Trp	Amino acid and Its metabolites	(1.77)	down
24	1-Methylhistidine	Amino acid and Its metabolites	(1.66)	down
25	Asn-Pro	Amino acid and Its metabolites	(1.61)	down
26	Pro-Asn	Amino acid and Its metabolites	(1.61)	down
27	Trp-Asp	Amino acid and Its metabolites	(1.54)	down
28	Glu-Leu	Amino acid and Its metabolites	(1.39)	down
29	Gly-Trp	Amino acid and Its metabolites	(1.35)	down
30	Glu-Tyr	Amino acid and Its metabolites	(1.34)	down
31	Lys-Phe	Amino acid and Its metabolites	(1.34)	down
32	Lys-Leu	Amino acid and Its metabolites	(1.29)	down
33	Allantoic acid	Amino acid and Its metabolites	(1.28)	down
34	N-Amidino-L-Aspartate	Amino acid and Its metabolites	(1.27)	down
35	L-Serine	Amino acid and Its metabolites	(1.11)	down
36	S-Allyl-L-cysteine	Amino acid and Its metabolites	(1.10)	down
37	Ser-Ser	Amino acid and Its metabolites	(1.09)	down
38	gamma-Glu-Phe	Amino acid and Its metabolites	(1.09)	down

39	Ile-Trp	Amino acid and Its metabolites	(1.07)	down
40	L-Homocitrulline	Amino acid and Its metabolites	(1.07)	down
41	N-(3-Hydroxypropyl)phthalimide	Benzene and substituted derivatives	(1.61)	down
42	3-Amino-2-naphthoic acid	Benzene and substituted derivatives	(1.57)	down
43	4-hydroxybenzylamine	Benzene and substituted derivatives	(1.53)	down
44	Oroxylin A	Benzene and substituted derivatives	(1.05)	down
45	4-methyl-5-thiazole-ethanol	Alcohol and amines	2.06	up
46	Bis(1-inositol)-3,1'-phosphate 1-phosphate	Alcohol and amines	1.51	up
47	Inositol 1-phosphate	Alcohol and amines	1.35	up
48	p-Hydroxyphenylethanolamine	Alcohol and amines	(3.15)	down
49	3-Methylthiopropylamine	Alcohol and amines	(1.74)	down
50	3-Epideoxycholic acid	Bile acids	(1.04)	down
51	Thiamine	CoEnzyme and vitamins	2.26	up
52	LPC(O-14:1)	GP	1.50	up
53	LPC(24:6e)	GP	1.28	up
54	LPE(20:5/0:0)	GP	1.14	up
55	LPC(24:6/0:0)	GP	1.09	up
56	LPC(16:2/0:0)	GP	1.08	up
57	LPC(17:2/0:0)	GP	1.06	up
58	LPE(0:0/20:5)	GP	1.03	up
59	LPE(16:1/0:0)	GP	1.00	up
60	LPC(22:3)	GP	(2.01)	down
61	LPC(O-18:2)	GP	(1.73)	down
62	LPE(0:0/20:2)	GP	(1.71)	down
63	LPE(22:3/0:0)	GP	(1.42)	down
64	LPE(20:1/0:0)	GP	(1.07)	down
65	Uridine-5'-diphospho-N-acetylgalactosamine disodium salt	Nucleotide and Its metabolites	1.80	up
66	B-Pseudouridine	Nucleotide and Its metabolites	1.02	up
67	Guanosine 3',5'-Cyclic Monophosphate	Nucleotide and Its metabolites	(2.07)	down
68	N6-Succinyl Adenosine	Nucleotide and Its metabolites	(1.45)	down
69	Creatine phosphate	Nucleotide and Its metabolites	(1.21)	down
70	Biliverdin	Tryptamines,Cholines,Pigments	(3.26)	down
71	D-Glucose 6-Phosphate	Carbohydrates and Its metabolites	1.90	up
72	D-Mannose 6-phosphate	Carbohydrates and Its metabolites	1.90	up
73	D-Fructose 6-Phosphate-	Carbohydrates and Its metabolites	1.83	up

	Disodium Salt			
74	Glucaric acid	Carbohydrates and Its metabolites	1.22	up
75	Mucic Acid	Carbohydrates and Its metabolites	1.22	up
76	Sucrose 6′-monophosphate	Carbohydrates and Its metabolites	1.15	up
77	2-O-α-D-glucopyranosyl-L-ascorbic acid	Carbohydrates and Its metabolites	(3.50)	down
78	1,5-Anhydro-D-Glucitol	Carbohydrates and Its metabolites	(3.02)	down
79	Rhamnose	Carbohydrates and Its metabolites	(3.02)	down
80	Hydroxyphenyllactic acid	Organic acid and Its derivatives	1.08	up
81	Sulfoacetic acid	Organic acid and Its derivatives	1.04	up
82	Hypaphorine	Organic acid and Its derivatives	(3.73)	down
83	3-Methylsalicylic acid	Organic acid and Its derivatives	(3.30)	down
84	2-Hydroxy-4-(methylthio)butyric acid	Organic acid and Its derivatives	(2.00)	down
85	2-Amino-3-phosphonopropionic acid	Organic acid and Its derivatives	(1.56)	down
86	Shikimic Acid	Organic acid and Its derivatives	(1.47)	down
87	Methylmalonic Acid	Organic acid and Its derivatives	(1.25)	down
88	Succinic Acid	Organic acid and Its derivatives	(1.25)	down
89	2-Aminoethanesulfinic Acid	Organic acid and Its derivatives	(1.24)	down
90	Guanidineacetic Acid	Organic acid and Its derivatives	(1.08)	down
91	Aminomalonic Acid	Organic acid and Its derivatives	(1.00)	down
92	2-Picoline	Heterocyclic compounds	1.12	up
93	Indoxylsulfuric acid	Heterocyclic compounds	(3.09)	down
94	Methoxyindoleacetic Acid	Heterocyclic compounds	(1.60)	down
95	N'-Methyl-2-pyridone-5-carboxamide	Heterocyclic compounds	(1.27)	down
96	Indole-3-lactic acid	Heterocyclic compounds	(1.09)	down
97	FFA(18:4)	FA	1.64	up
98	Carnitine C12:1-2OH	FA	1.57	up
99	Carnitine C18:4	FA	1.36	up
100	Carnitine ph-C1	FA	1.32	up
101	(±)12-HETE	FA	1.13	up
102	Carnitine C5:0	FA	(1.05)	down
103	Carnitine-2-methyl-C4	FA	(1.05)	down

Note: CK - control group, T - treatment group with the addition of ε - polylysine and antioxidant, the number indicate storage day. The numbers in parentheses are negative values.

Table S5. Differential metabolite of T7 vs. CK7 with both VIP and absolute value of Log2FC > 1

	Compounds	Class I	Log2FC	Type
1	N-Acetylthreonine	Amino acid and Its metabolites	6.12	up
2	Phe-Met	Amino acid and Its metabolites	1.40	up
3	Phe-Ala-Ser	Amino acid and Its metabolites	1.27	up
4	Ser-Phe-Ala	Amino acid and Its metabolites	1.27	up
5	Phe-Ser	Amino acid and Its metabolites	1.22	up
6	Ser-Phe	Amino acid and Its metabolites	1.22	up
7	Val-Val	Amino acid and Its metabolites	1.21	up
8	Tyr-Ala	Amino acid and Its metabolites	1.20	up
9	Ala-Tyr	Amino acid and Its metabolites	1.16	up
10	Val-Ile	Amino acid and Its metabolites	1.10	up
11	Val-Leu	Amino acid and Its metabolites	1.10	up
12	Met-Phe	Amino acid and Its metabolites	1.10	up
13	N-lactoyl-phenylalanine	Amino acid and Its metabolites	1.00	up
14	L-Theanine	Amino acid and Its metabolites	(2.61)	down

15	Nα-Acetyl-L-Arginine	Amino acid and Its metabolites	(2.54)	down
16	γ-Glu-Met	Amino acid and Its metabolites	(1.82)	down
17	Lys-Phe	Amino acid and Its metabolites	(1.75)	down
18	Allantoic acid	Amino acid and Its metabolites	(1.73)	down
19	Thr-Trp	Amino acid and Its metabolites	(1.68)	down
20	S-Sulfo-L-Cysteine	Amino acid and Its metabolites	(1.62)	down
21	2-amino-6-oxohexanoic acid	Amino acid and Its metabolites	(1.62)	down
22	1-Methylhistidine	Amino acid and Its metabolites	(1.61)	down
23	Cys-Gly	Amino acid and Its metabolites	(1.56)	down
24	L-Cystine	Amino acid and Its metabolites	(1.38)	down
25	Trp-Asp	Amino acid and Its metabolites	(1.37)	down
26	Glu-Leu	Amino acid and Its metabolites	(1.36)	down
27	N-Amidino-L-Aspartate	Amino acid and Its metabolites	(1.28)	down
28	γ-Glu-Cys	Amino acid and Its metabolites	(1.25)	down
29	Asn-Pro	Amino acid and Its metabolites	(1.21)	down
30	Pro-Asn	Amino acid and Its metabolites	(1.21)	down
31	L-Homocitrulline	Amino acid and Its metabolites	(1.15)	down
32	S-Allyl-L-cysteine	Amino acid and Its metabolites	(1.13)	down
33	Glu-Tyr	Amino acid and Its metabolites	(1.02)	down
34	N-Acetylaspartate	Amino acid and Its metabolites	(1.00)	down
35	4-acetoxyphenol	Benzene and substituted derivatives	3.07	up
36	4-(Methylamino)phenol	Benzene and substituted derivatives	(1.54)	down

37	3-Amino-2-naphthoic acid	Benzene and substituted derivatives	(1.51)	down
38	Dipyrocetyl	Benzene and substituted derivatives	(1.48)	down
39	N-(3-Hydroxypropyl)phthalimide	Benzene and substituted derivatives	(1.47)	down
40	4-hydroxybenzylamine	Benzene and substituted derivatives	(1.35)	down
41	Bis(1-inositol)-3,1'-phosphate 1-phosphate	Alcohol and amines	1.29	up
42	Inositol 1-phosphate	Alcohol and amines	1.24	up
43	p-Hydroxyphenylethanolamine	Alcohol and amines	(3.44)	down
44	3-Methylthiopropylamine	Alcohol and amines	(1.62)	down
45	Triethylenetetramine	Alcohol and amines	(1.08)	down
46	LPC(O-14:1)	GP	1.59	up
47	LPC(24:6/0:0)	GP	1.34	up
48	LPC(24:6e)	GP	1.10	up
49	LPC(22:3)	GP	(2.96)	down
50	LPC(O-18:2)	GP	(1.85)	down
51	LPE(22:3/0:0)	GP	(1.83)	down
52	LPE(0:0/20:2)	GP	(1.52)	down
53	Arachidonoyl LPA	GP	(1.34)	down
54	LPE(20:2/0:0)	GP	(1.16)	down
55	MG(22:5/0:0/0:0)	GL	1.20	up
56	MG(0:0/22:6/0:0)	GL	1.17	up
57	MG(22:6/0:0/0:0)	GL	1.17	up
58	Xanthine	Nucleotide and Its metabolites	2.62	up
59	Uridine-5'-diphospho-N-acetylgalactosamine disodium salt	Nucleotide and Its metabolites	1.72	up
60	Creatine phosphate	Nucleotide and Its metabolites	(1.72)	down
61	N6-Succinyl Adenosine	Nucleotide and Its metabolites	(1.41)	down
62	GDP-L-fucose	Nucleotide and Its metabolites	(1.36)	down

63	2'-Deoxycytidine-5'-Monophosphate	Nucleotide and Its metabolites	(1.32)	down
64	Theobromine	Nucleotide and Its metabolites	(1.01)	down
65	Diethyl sebacate	Aldehyde,Ketones,Esters	1.00	up
66	Biliverdin	Tryptamines,Cholines,Pigments	(3.56)	down
67	D-Glucose 6-Phosphate	Carbohydrates and Its metabolites	1.67	up
68	D-Mannose 6-phosphate	Carbohydrates and Its metabolites	1.67	up
69	D-Fructose 6-Phosphate-Disodium Salt	Carbohydrates and Its metabolites	1.56	up
70	Sucrose 6′-monophosphate	Carbohydrates and Its metabolites	1.15	up
71	Glucaric acid	Carbohydrates and Its metabolites	1.03	up
72	Mucic Acid	Carbohydrates and Its metabolites	1.03	up
73	2-O-α-D-glucopyranosyl-L-ascorbic acid	Carbohydrates and Its metabolites	(3.87)	down
74	1,5-Anhydro-D-Glucitol	Carbohydrates and Its metabolites	(1.15)	down
75	Rhamnose	Carbohydrates and Its metabolites	(1.15)	down
76	Hydroxyphenyllactic acid	Organic acid and Its derivatives	1.33	up
77	Sulfoacetic acid	Organic acid and Its derivatives	1.03	up
78	Hypaphorine	Organic acid and Its derivatives	(3.82)	down
80	2-Hydroxy-4-(methylthio)butyric acid	Organic acid and Its derivatives	(2.91)	down
81	2-Amino-3-phosphonopropionic acid	Organic acid and Its derivatives	(1.69)	down
82	Methylmalonic Acid	Organic acid and Its derivatives	(1.67)	down
83	Succinic Acid	Organic acid and Its derivatives	(1.67)	down
84	EDTA	Organic acid and Its derivatives	(1.60)	down
85	Aminomalonic Acid	Organic acid and Its derivatives	(1.42)	down

86	3-Methylsalicylic acid	Organic acid and Its derivatives	(1.38)	down
87	2-Aminoethanesulfinic Acid	Organic acid and Its derivatives	(1.33)	down
88	2-O-Methylcytosine	Heterocyclic compounds	1.49	up
89	Indoxylsulfuric acid	Heterocyclic compounds	(2.72)	down
90	Methoxyindoleacetic Acid	Heterocyclic compounds	(1.48)	down
91	Indole-3-lactic acid	Heterocyclic compounds	(1.11)	down
92	Carnitine C18:4	FA	1.85	up
93	FFA(18:4)	FA	1.63	up
94	Carnitine C10:2	FA	1.45	up
95	Carnitine ph-C1	FA	1.31	up
96	(±)12-HETE	FA	1.22	up
97	Carnitine C5:0	FA	(1.00)	down
98	Carnitine-2-methyl-C4	FA	(1.00)	down

Note: CK - control group, T - treatment group with the addition of ε - polylysine and antioxidant, the number indicate storage day. The numbers in parentheses are negative values.
